# Waste Refinery: The Valorization of Waste Plastics and End-of-Life Tires in Refinery Units. A Review

**DOI:** 10.1021/acs.energyfuels.0c03918

**Published:** 2021-02-09

**Authors:** Roberto Palos, Alazne Gutiérrez, Francisco J. Vela, Martin Olazar, José M. Arandes, Javier Bilbao

**Affiliations:** Department of Chemical Engineering, University of the Basque Country UPV/EHU, PO Box 644, 48080 Bilbao, Spain

## Abstract

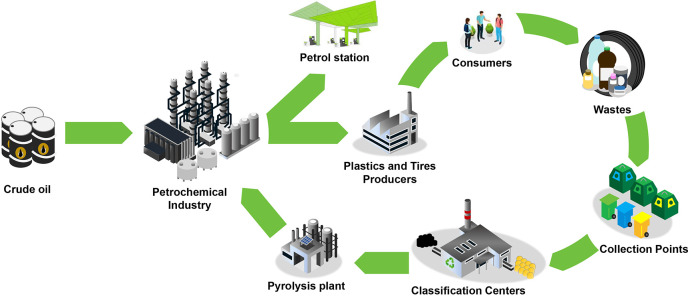

This review collects a wide range of initiatives and results that expose the potential of the refineries to be converted into waste refineries. Thus, they will use their current units for the valorization of consumer society wastes (waste plastics and end-of-life tires in particular) that are manufactured with petroleum derivatives. The capacity, technological development, and versatility of fluid catalytic cracking (FCC) and hydroprocessing units make them appropriate for achieving this goal. Polyolefinic plastics (polyethylene and polypropylene), the waxes obtained in their fast pyrolysis, and the tire pyrolysis oils can be cofed together with the current streams of the industrial units. Conventional refineries have the opportunity of operating as waste refineries cofeeding these alternative feeds and tailoring the properties of the fuels and raw materials produced to be adapted to commercial requirements within the oil economy frame. This strategy will contribute in a centralized and rational way to the recycling of the consumer society wastes on a large scale. Furthermore, the use of already existing and, especially, depreciated units for the production of fuels and raw materials (such as light olefins and aromatics) promotes the economy of the recycling process.

## 1 Introduction

Growing population is the root cause of the progressive damage to the environment. The production of waste in the consumer society is directly related to the development and improvement of the standard of living. However, it has also caused one of the greatest environmental issues that paradoxically is a threat to human development. Furthermore, the following factors tend to aggravate the current environmental situation:^1^ (i) replacement of traditional materials by plastic materials (packaging, building and construction, furniture, utensils, etc.); (ii) increase and concentration of population (7.7 billion in 2020 and a forecast of 9.7 billion in 2050 with a migration rate from the countryside to the cities that has increased from 30% in 1950 to 55% in 2019); (iii) global access to consumer society increasing the use of plastics, together with the acquisition, replacement, and disposal of tires. Different environmental reports that have a great impact on public opinion, such as those that analyze CO_2_ emissions, global warming, or the presence of microplastics in the oceans, together with the shocking events caused by the COVID-19 pandemic, have brought the conviction that human health care and wellness requires the conservation of the environment. Within this scenario, new agreements and laws are adopted to reduce waste generation and to manage waste, establishing political interventions to strengthen the culture of environmental protection and recycling. Hence, the adoption of 5R principles (reduce, reprocess, reuse, recycle, and recover) and the use of renewable resources has been consolidated in the daily life of the citizens and regulates the actuation of every industrial activity according to the Circular Economy.

The mechanical recycling of tires and plastics and the incorporation of mechanically recycled materials alongside virgin resins into production processes have severe limitations. Thus, the repolymerization process is affected by the lack of stability of the materials leading to a reduction in the quality of the products obtained. Moreover, these solutions cannot be applied on a large scale.^2^ The valorization routes of highest viability for these wastes are the thermochemical processes. Among them, pyrolysis, either thermal or catalytic, is the one with the highest expectations for the production of fuels and chemicals because of the notable technological development it has undergone.^3^ Nevertheless, the establishment of new industries for the production of fuels and raw materials from waste plastic and discarded tires has to face technological and economic difficulties, apart from those involving the production of high-quality products suitable to be added to the well-established oil market.

The recycling of end-of-life (EOL) consumer goods, such as the plastics and tires produced from petroleum-derivative chemicals, can be faced by the oil industry. This review gathers different research initiatives that propose the valorization of these wastes in two conventional refinery units, fluid catalytic cracking and hydroprocessing units. Focus has been specifically placed on the valorization of plastics (polyolefins) dissolved in current refinery streams and on the valorization of the liquid products obtained in the fast pyrolysis of polyolefins (plastic pyrolysis oil, PPO) and of EOL tires (tire pyrolysis oil, TPO). The results are evidence of the potential capacity of refinery units (waste refinery) for the large-scale recycling of waste plastics and EOL tires and contribute to solving the severe environmental issues derived from their mismanagement.

Tables 1 and 2 summarize the main items of some of the numerous reviews that can be found in the literature about the different thermochemical routes available for the valorization of waste plastics and EOL tires, respectively. These reviews have mainly focused on the fast pyrolysis of the wastes, and their principal scopes have been the different reactor types, operating conditions, and types of catalysts used, together with their effects on the yields and composition of the obtained products. The product that has attracted most attention in the literature has been the liquid one (PPO and TPO) because of its possible use as a fuel. It is worth noting that the main topics studied in these reviews about the pyrolysis of waste plastics and EOL tires are complementary to those of this review, the originality of which lies on the possible integration of the fast pyrolysis units with conventional refinery units.

**Table 1:** Reviews about the Thermochemical Routes for the Valorization of Waste Plastics

reference	main items
**Thermal and Catalytic Pyrolysis**	
Wong et al.^4^	different technologies for the production of fuels
	fuels of single type plastics, mixed and municipal waste plastics
	
Anuar Sharuddin et al.^5^	different technologies and operating conditions
	composition and properties of the gas the liquids products to be used as fuels
	
Al-Salem et al.^6^	reaction technologies
	role of the catalyst in the pyrolysis
	
Lopez et al.^3^	technologies and operating conditions for the production of fuels and raw materials from different plastics
	pros and cons of each technology
	
Kasar et al.^7^	reaction technologies
	effects of the operating conditions on obtained products
	co-pyrolysis of plastics with oil-derived residues
	
Qureshi et al.^8^	opportunities and challenges for the commercialization of the liquid product as a fuel
	
Solis and Silveira^9^	pros and cons of the thermochemical routes
	degree of establishment of different commercial technologies and pilot plants
	
**Catalytic Pyrolysis**	
Serrano et al.^10^	effects of the porous structure and acidity of the catalyst on the product distribution obtained in the cracking of polyolefins
	
Miandad et al.^11^	advantages of catalytic pyrolysis
	catalysts for the pyrolysis of different plastics
	effects of the catalyst on the product composition and distribution
	
Li et al.^12^	different catalysts in the pyrolysis of municipal solid wastes (mixtures of plastics, paper, textiles, organic wastes, and others)
	
Mark et al.^13^	analysis of the performance of different catalysts for the cracking of plastics

**Table 2:** Reviews about the Thermochemical Routes for the Valorization of EOL Tires

reference	main items
**Thermochemical Routes**	
Rowhani and Rainey^14^	management technologies and conditions
	pyrolysis technologies
	effects of the reactor type, operating conditions, and catalyst type on the product distribution
	
**Thermal Pyrolysis**	
Antoniou et al.^15^	policy and legislative issues in the EU
	reactor configurations (bench, pilot, and industrial scales)
	composition of obtained products
	economical, energetic, and environmental analysis
	
Martínez et al.^16^	investigations and patents
	advantages of pyrolysis
	effects of the reactor type and operating conditions on the product composition and distribution
	
Williams^17^	reactors and commercial and semicommercial plants
	effects of the operating conditions on the composition of the liquid product
	properties as a fuel of the liquid product
	composition of the gas and solid products
	
Sathiskumar and Karthikeyan^18^	valorization routes of the liquid product: as a fuel or as a source of BTX and limonene
	valorization of the gas and solid products (pyro-gas and pyro-char)
	
Czajczyńska et al.^19^	effects of the operating conditions on the composition of obtained products
	environmental impact of the composition (nitrogen, sulfur, and metals)
	
**Uses of the Liquid Product**	
Januszewicz et al.^20^	analysis of different reactor types and of the operating conditions for maximizing the yield of limonene
	
Zhang et al.^21^	analysis of the composition and properties of the liquid product
	separation of the limonene
	possible use of the liquid product as a fuel
	synthesis of carbon material and bitumen
	
**Uses of the Solid Product**	
Xu et al.^22^	rubber manufacture, as activated carbon and as biochar for soil improvement
	
Okoye et al.^23^	carbon black production mechanisms
	perspectives of using pyrolysis liquid product for carbon black manufacturing
	
**Catalytic Pyrolysis**	
Arabiourrutia et al.^24^	different pyrolysis technologies
	reaction mechanisms
	effects of the reactor type, operating conditions, and properties of the catalyst on the product distribution and composition

## 2 Petroleum Derivative Wastes in the Consumer Society

Among the different types of wastes that can be found in the municipal solid waste (MSW), the ones that attract greatest attention are waste plastics and EOL tires, as hydrocarbons and chemicals produced in refineries are used in their manufacturing. Consequently, their recycling is potentially feasible in a refinery.

### 2.1 Waste Plastics

#### 2.1.1 Generation

**Figure 1:**
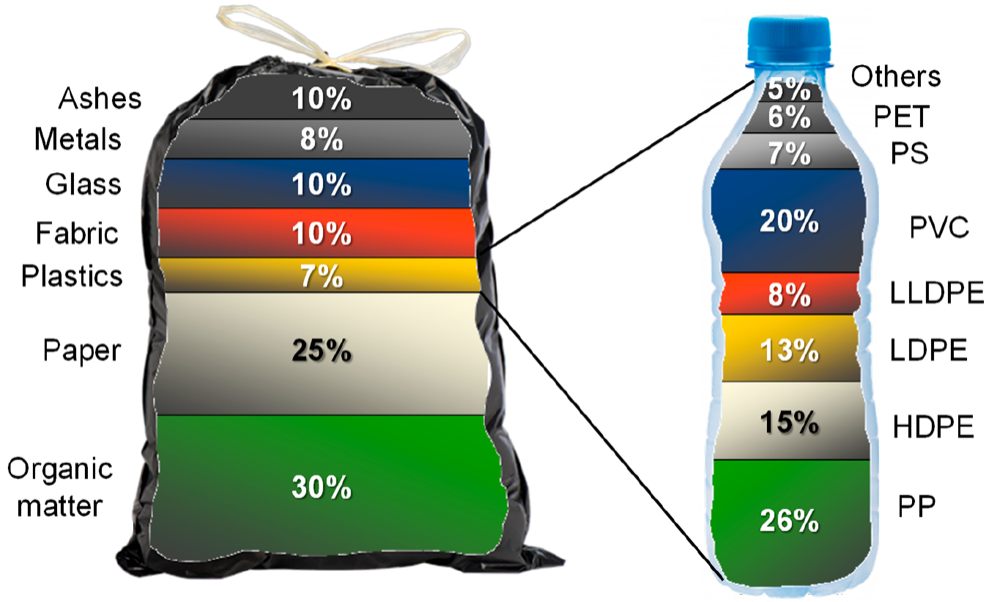
Average composition (wt %) of MSW in the EU and of its plastics fraction.

Given the non-biodegradability of plastics and tires and their contribution to the total amount of wastes disposed in landfills, their increasing generation is becoming a serious problem. The production of plastics has increased steadily since their first appearance in the market in the 1930s reaching 359 million tons produced worldwide in 2018 (348 million tons in 2017). Asia is the region that produced the largest amount of plastics, 51% of the total amount (30% China, 4% Japan), whereas North America and the EU produced 18% and 17%, respectively.^25^ This historical development is explained by their low manufacturing costs and excellent properties for multiple applications in different areas. Postconsumer waste plastics stem from five big sectors: agriculture, automotive, building and construction, distribution, and packaging. A more detailed study shows that agriculture, automotive, building and construction, and distribution sectors account for the generation of 40% of the plastic wastes, whereas the remaining 60% derives from the packaging sector. This last group is the main plastic fraction found within MSW. The average MSW composition in EU is detailed in Figure 1, where it can be seen that plastics only account for 7 wt % of the trash. Polyolefins (PP, HDPE, LDPE, and LLDPE) are the main plastic types (>60 wt %) and PVC, PS, and PET also appear in considerable concentrations.^26^ However, because of their low density, the volume contribution of the plastics to the MSW increases to 20 vol %. Accordingly, on the basis of 1.35 kg of municipal waste generated per person and day, around 19 million tons of the 270 million tons of MSW collected in the EU in 2019 are plastics.^25^ Moreover, a fact to be highlighted is the huge increase in the use of health care materials, personal protective equipment, and single-use plastics in 2020 due to the COVID-19 pandemic, which undoubtedly will contribute to increasing the generation of waste plastics.^27^

#### 2.1.2 Management

Even though only 8% of the oil consumed worldwide can be attributed to the plastics industry,^28^ the interest in their recycling is based on the need to reduce their disposal in landfills. This is a consequence of their low biodegradability, as the lifetime of most of the plastic wastes ranges from 1 to 35 years.^29^ Geyer et al.^30^ estimated that the worldwide waste plastics production up to 2015 accounted for 6300 million tons, of which 9% have been recycled, 12% incinerated, and the remaining 79% accumulated in landfills or in natural environments. These authors also estimated that, without significant recycling efforts, 12 000 million tons of waste plastics might be disposed by the year 2050. Furthermore, the waste plastics disposed in landfills undergo gradual fragmentation into microplastics (MPs, particles of <5 mm diameter) through mechanical and microbial decomposition, weathering, photolysis, and abrasion. This phenomenon, together with the release of manufactured MPs contained in various consumer goods (microbeads, capsules, fibers, or pellets in cosmetics, personal care products, cleaning agents, paints, and coatings) are the main contributors to the 243 000 tons of MPs afloat in the oceans.^31^ The high surface area and hydrophobicity of these materials ease their ingestion by living organisms and promote the risk of adsorption and desorption of toxic chemicals and pathogens in water. Accordingly, it is well established that the presence of MPs in aquatic organisms has negative health effects, such as growth and development inhibition, neurotoxic responses, metabolic disorders, and genotoxicity.^32,33^ Likewise, the presence of MPs in the soil also affects its properties, plant performance, and microbial activities.^34^ Moreover, the inhalation of smaller MPs (nanoplastics, NPs) and the ingestion of MP/NP-containing foodstuffs by human beings (ultimate consumers in the food chain) may involve potential risks, whose dependency on the composition and concentration of MPs/NPs is still under study.^35^

Apart from being landfilled, waste plastics can be also incinerated in order to produce energy or recycled to recover the monomers that they contain. These disposal methods were of low significance before 1980. From 1980 and 1990 onward, incineration and recycling rates have increased an average of 0.7% per year, reaching average values of 28.3% and 19.3%, respectively, in the year 2019.^30,36^ However, energy recovery or recycling rates greatly change depending on the country or region.^37^ Incineration of waste plastics is the main disposal method in various countries. Thus, Japan, Sweden, and Denmark incinerate 56, 81.7, and 57.1 wt % of the plastics, respectively, with the aim of recovering energy. This activity is carried out by taking severe measures to control emissions.

As plastics are final petroleum products, it seems logical to associate their recycling with the petrochemical industry and the production of chemicals. Waste plastics could be reintroduced in different manufacturing stages, by means of primary, secondary (mechanical), or tertiary (chemical) recycling. Among the different recycling routes, those with higher prospects to be implemented on a large scale are the thermochemical routes of tertiary recycling. These routes allow the production of fuels and the recovery of the monomers, which may be converted into the original material from which they came. Different reviews of these thermochemical routes have already been reported focusing on the initiatives associated with pyrolysis^3^ and gasification^38^ of waste plastics.

### 2.2 EOL Tires

#### 2.2.1 Generation

**Figure 2:**
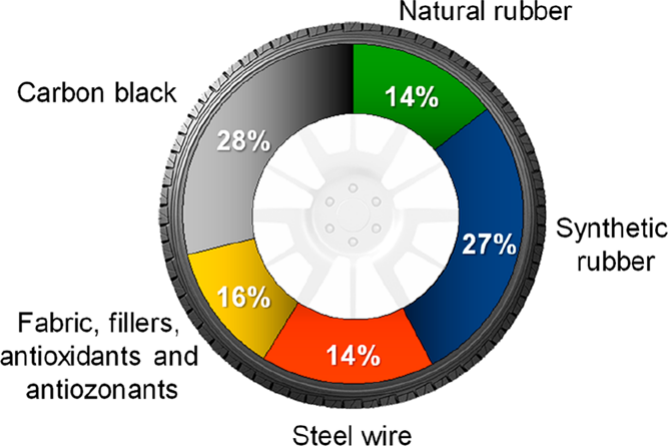
Average composition of a passenger tire by weight.

Tires are products of complex engineering. They are the result of the assembly of more than 200 components. Among the components used in the manufacturing of tires, rubber (both natural and synthetic), carbon black, inert fillers (amorphous precipitated silica, alumina), steel, textile and fabric cords (nylon, kevlar), sulfur, zinc oxide, and different antioxidants and antiozonants are the main ones. Figure 2 shows the average composition of passenger tires,^39^ which depends on the type of vehicle (cars, trucks, buses, planes, etc.) and regional climatology. Because of its complex composition and structure, once a tire reaches the end of its lifespan, it cannot be restored and directly reused. Hence, it becomes an EOL tire. Furthermore, the presence of different components creates difficulties for their recycling.

The International Rubber Study Group has estimated that 14.8 million tons of rubber were consumed in 2019 all over the world, with 60% being used for the manufacturing of tires. It should be added that the manufacturing of each tire consumes between 23.5 and 141 L of oil,^40^ which is evidence of the high usage of resources involved in this industry. Furthermore, estimations account for an average production of 17 million tons of EOL tires per year, which means that about 2800 million tires are disposed every year (assuming that an average tire weights 6 kg).^41^ Therefore, the notable increase in the worldwide motorization rate and so in tire consumption surpasses the impact of the measures designed to extend their life cycle.^42,43^ Furthermore, both the dumping and disposal in landfills of EOL tires may cause (i) groundwater pollution, (ii) uncontrolled and hazardous fires with high levels of emissions of carbon monoxide, nitrogen and sulfur oxides, volatile organic compounds (VOCs), polycyclic aromatic hydrocarbons (PAHs), and heavy metals, and (iii) proliferation of rodents, mosquitos, or termites.

#### 2.2.2 Management

The manufacturing of tires involves irreversible vulcanization processes. In these processes, the layers of synthetic and natural rubber, sulfur, and other components are cross-linked conferring elasticity, insolubility, and infusibility upon the tire.^44^ Consequently, the recovery of materials and chemicals from discarded tires requires energy demanding processes involving mechanical, thermal, or chemical destruction of the rubber.^45^ The trends observed in the management of EOL tires over the last 20 years have consisted in a slight increase in the routes involving material recovery and a reduction of those for energy recovery, with reuse being steady and gradually reduced.^46^

Energy recovery is a relevant route for EOL tire management provided that environmental impacts are under control.^47,269^ Advantages involving the use of this waste in the ovens of ceramic and cement factories are as follows:^48,49^ (i) saving of raw materials, electricity, and fuels; (ii) mitigation of CO_2_ emissions due to the high content of rubber in the tires; (iii) the possibility of cofeeding with other wastes without affecting the efficiency of the oven. Similarly, Rowhani and Rainey^14^ have enumerated some advantages of EOL tire incineration: (i) the possibility of producing electricity and steam and (ii) the recovery of several raw materials used in the manufacturing of the tires, such as steel wires, zinc oxide, and sodium sulfate. Based on these facts, the tire industry approached the incineration of EOL tires in rotary kilns with the aim of producing steam for the vulcanization process and of reducing the environmental impact of this waste.^51^

According to the European Tire and Rubber Manufacturers’ Association,^52^ from the 3.5 million tons of EOL tires that were generated in 2018 in the EU, 91% were collected and treated for material recycling and energy recovery. About 1.2 million tons of EOL tires (35% of the amount generated) were treated through energy recovery, especially in cement kilns (75%) and urban heating and power plants (25%). The remaining 2 million tons (53% of total EOL tires generated) were used for material recovery purposes, including granulation (78%) and their use in civil engineering and public works (about 5%). With regard to the USA, just the 72.9% of the EOL tires produced were collected and treated in 2019 according to the U.S. Tire Manufacturers Association.^53^ Furthermore, the amount of tires treated through energy recovery and recycled were similar, the former being slightly higher (38.2% and 34.7%, respectively). The remaining 27.1% of tires discarded in the USA suffer the worst destiny, as they are legally and illegally land disposed (14.3% and 9.7%, respectively) or exported to other countries (3.1%).

Therefore, in concordance with the aforementioned data about the low recycling level and the low added value of obtained products, the generation of plastic waste and EOL tires far exceeds the capacity of the currently established management routes. This fact promotes the development of new valorization routes suitable to be implemented on large scale with the required economic viability. Accordingly, thermochemical routes are the most promising ones, specially gasification and pyrolysis.

## 3 The Concept of Waste Refinery

As previously stated, important advances have been made in the technologies for tertiary recycling of waste plastics and EOL tires, with emphasis being placed on the development of pyrolysis technology (Section 4) for the production of fuels and the recovery of monomers. Nonetheless, there is no industrial initiative for the valorization of these wastes with the required capacity to solve the current mismanagement. This situation is strongly affected by the following drawbacks: (i) a big economic investment is required for the implementation of the units required for the integral valorization of these wastes at large scale; (ii) the obtained products must meet severe quality standards established by current legislation; (iii) this new and alternative industry will have to compete with the well-established oil industry. Consequently, the situation suggests the promotion of a large-scale waste valorization industry (waste refinery) by integrating primary waste valorization units within refineries. Accordingly, primary units will produce low-quality streams that will be converted into fuels and commodities (light olefins and aromatics) in the large-scale secondary treatment units available in refineries.

The waste refinery appears as a new concept given the necessity to make technologically and economically viable the large-scale valorization routes of waste plastics and EOL tires. It can be defined as “*a plant that integrates conversion processes with units for the production of fuels, energy, and chemicals, either from wastes and their derivatives or from secondary refinery streams*”.

**Figure 3:**
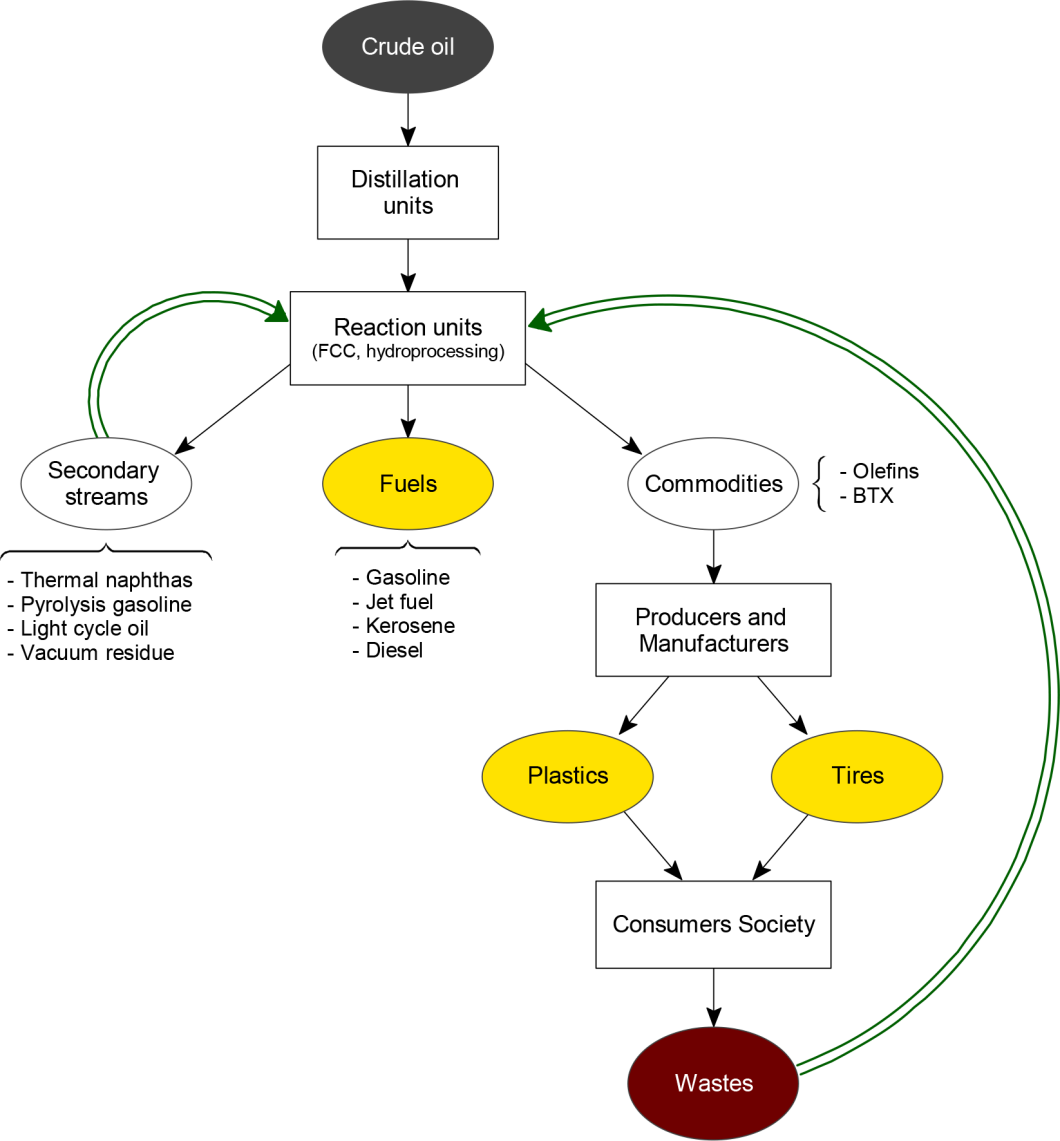
Scheme that describes the concept of waste refinery, which consists in the recycling of secondary refinery streams and petroleum-derived wastes.

Therefore, the numerous activities that a waste refinery brings together can be divided into two series of interrelated actions (Figure 3). The first series corresponds to the initiatives of the petroleum industry itself, as it generates secondary refinery streams as byproducts of distillation and reaction units. The processing of these side streams follows an increasing trend in refineries in order to intensify the valorization of oil by means of increasing the yield of commercial products. Indeed, the FCC unit plays a key role in the cofeeding of vacuum residue^54^ and of visbreaker and coker heavy naphthas.^55−59^ Equally, hydroprocessing units can be appropriate for the cofeeding of aromatic streams, such as the pyrolysis gasoline obtained in steam cracker units^60^ or the light cycle oil (LCO) obtained in FCC units,^61−63^ with the aim of producing naphtha and medium distillates or BTX aromatics.^64^ The second series of actions of the waste refinery, which constitutes the interest of this review, focuses on the recycling of consumer society wastes, for example, waste plastics and EOL tires. Recycling activities relate refinery units with other additional units, which will develop the ecoindustry. Among the required additional units, the one for pyrolysis is key for the conversion of waste solids into liquid streams that can be fed into refinery units, either as they are produced or blended with common feeds.

Based on their versatility, the refinery units with higher prospects for managing these feeds (raw plastics, waste plastic pyrolysis oil, and EOL tire pyrolysis oil) are the following ones: catalytic cracking (FCC), hydroprocessing, steam cracking, and coker units.^54,65,66^ Moreover, taking into account their capacity and technological development, the refinery units that forge ahead in the implementation of the waste refinery are the FCC unit (in the short term, using already depreciated units) and the hydroprocessing unit (in the long term, given its higher complexity and lower implementation). Next, in sections 5 and 6, the main features of these units have been summarized, together with the main research results obtained in the catalytic cracking and hydroprocessing of these wastes. Furthermore, a refinery is equipped with separation, purification, and other units appropriate for the integral valorization of the remaining streams of products obtained in the pyrolysis of waste plastics and EOL tires, such as light olefins and BTX aromatics.

The oil industry is immersed in a big dilemma given the change in the energy model society is demanding and the fluctuations in the availability, quality, and price of crude oil.^67^ Within this scenario, the involvement of the refineries in waste recycling may be boosted by economic incentives and subsidies provided by public administration, which will undoubtedly help to finance the revamping of the FCC and hydroprocessing units. Moreover, global emissions of CO_2_ will be notably reduced entailing a reduction in the carbon taxes of the corresponding country. Furthermore, the contribution of the oil industry to resolve an urgent environmental issue as that involving the uncontrolled disposal of these wastes would help to improve the image and projection of oil refineries.

**Figure 4:**
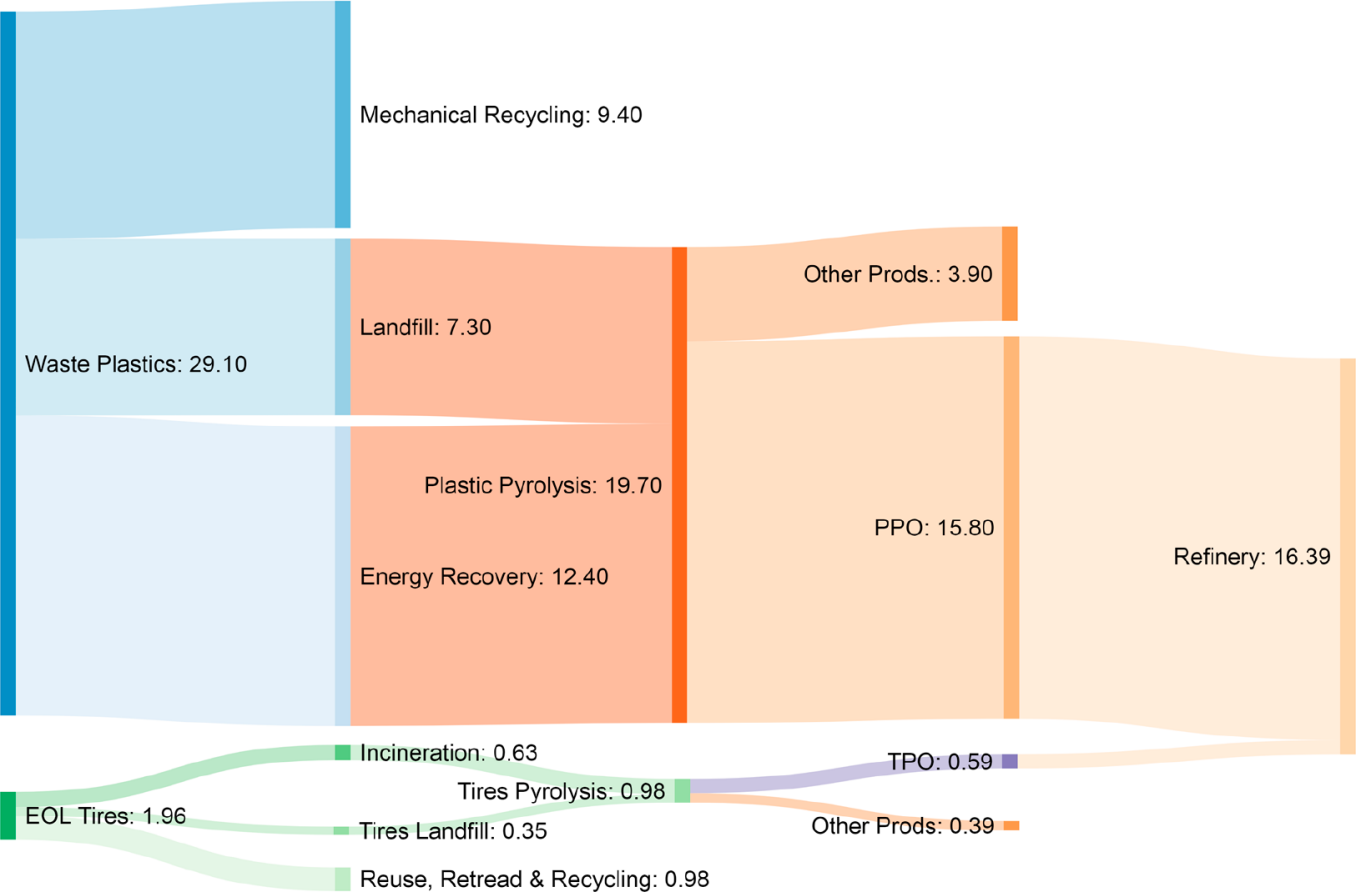
Availability of hydrocarbons for refineries (in million tons) if EU waste plastics and EOL tires were managed according to the model proposed by waste refinery.

Furthermore, oil refineries may save an important amount of crude oil by recycling the waste plastics and EOL tires. Figure 4 computes the total amount of hydrocarbons that can be obtained from these wastes in the EU. Therefore, analyzing the case of waste plastics first, 29.1 million tons were generated in 2018 in the EU.^25^ From this amount, 9.4 million tons were mechanically recycled, whereas the remaining 19.7 million tons were landfilled or burned for energy recovery. Thus, assuming that neither landfilling nor combustion are the optimal management routes, these wastes may have been pyrolyzed. Taking into account that waste plastic pyrolysis might lead to liquid yields of 80 wt %,^68^ an amount of 15.8 million tons of plastic pyrolysis oil (PPO) suitable for treatment in refinery units might have been produced. Note that from the total amount of PPO produced, two-thirds approximately correspond to the PPO obtained from polyolefins. Likewise, the same analysis can be performed for the EOL tires. Thus, 1.96 million tons of EOL tires were produced in the EU in 2018. Half of these were incinerated (0.63 million tons) or landfilled (0.35 million tons). If the 0.98 million tons of mismanaged EOL tires had been submitted to a pyrolysis stage, 0.59 million tons of tire pyrolysis oil (TPO) would have been produced assuming a liquid yield of 60 wt %.^69^ Consequently, a total amount of 16.39 million tons of hydrocarbons would have been available for European refineries, which means an important source of raw materials considering that 740 million tons of crude oil are processed on average in the EU.^70^

## 4 Fast Pyrolysis

The generation of plastic waste and EOL tires far exceeds the capacity of currently established management routes. This fact and the limitations derived from the environmental restrictions on incineration promote the development of new valorization routes suitable to be implemented at large scale with the required economic viability. Thermochemical routes are the most promising ones, specially fast pyrolysis, because the liquid and gaseous products obtained may be valorized in line or in subsequent catalytic stages. Pyrolysis (or thermal cracking) requires high temperatures and is commonly carried out in non-oxidizing atmospheres (in the absence of O_2_). This process breaks down solid wastes into three different fractions: gas, liquid (oil), and solid (commonly known as char). The ratios of the different fractions obtained depend on the operating conditions, but especially on temperature and residence time of the volatiles. Fast pyrolysis is characterized by high heating rates and short residence time of the volatiles, which maximizes the yield of the oil obtained. Among the advantages of fast pyrolysis, those worth mentioning are as follows:^71^ (i) versatility, as wastes of different nature (agroforestry wastes, plastics, tires, sewage sludge) can be cofed;^72,73^ (ii) reduced environmental impact, as pyrolysis produces lower emissions than gasification.^74^ Moreover, pyrolysis can be performed under vacuum by reducing the gas flow rate,^75^ but it can be also carried out in autothermal regime by cofeeding O_2_.^76^ There are a variety of reactor configurations (moving, fluidized, or spouted beds) for continuous fast pyrolysis.^3^ Furthermore, their simple design makes feasible the manufacturing of smaller or movable units that may operate nearby waste collection and specific locations where *in situ* pyrolysis may be conducted. The energy requirements of the pyrolysis unit may be covered by the combustion of the gas fraction produced or a fraction of the input mass flow rate^77^ (on the order of 5 wt %) or by concentrated solar energy as an alternative.^78^ Finally, the interest for the waste refinery lies in the possibility of transporting the pyrolysis oil to the refinery and treating it on a large scale in the corresponding refinery unit.^3^

Even though the reviews summarized in Tables 1 and 2 collect a great part of the studies available in the literature about the pyrolysis of waste plastics and EOL tires, respectively, some works are discussed below, since they can contribute to understanding the state of the art of this thermochemical route. Indeed, the suitability of the refinery units for the valorization of the liquid product obtained from waste plastics and EOL tires (PPO and TPO, respectively) has been stressed.

**Table 3:** PONA Analysis Results of the PPO from IPW, PCPW, and PWPW

	composition (wt %)		
components	IPW	PCPW	PWPW
paraffins	38.7	50.5	57.8
olefins	18.4	22.5	19.3
naphthenes	16.5	19.0	14.2
aromatics	26.4	8.0	8.7

Adapted from the work by Gala et al.^97^

**Table 4:** Main Properties of an Average TPO, Commercial Gasoline, and Commercial Diesel

property	TPO	gasoline	diesel
density (kg m^–3^)	830	780	838
viscosity (cSt)	4.75		2.1
flash point (°C)	65	43	54
HHV (MJ kg^–1^)	42.7	43.9	45.5
elemental analysis (wt %)			
C	79.96	85	87.4
H	10.04	14.1	12.1
N	0.94	0.02	0.04
S	0.11	0.03	0.29
O	9.3		0.29
boiling points (°C)			
IBP	38.5	34	171.5
*T*_50_	174.8	92	265.6
*T*_90_		154	335.8
FBP	382.4	218	364.6

Adapted from the work by Rowhani and Rainey.^14^

### 4.1 Waste Plastics

Pyrolysis is an environmentally friendly option for managing plastic wastes, especially addition polymers, which are the main ones within the MSW. A goal extensively studied in the literature has been recovery of monomers (light olefins) by means of fast pyrolysis in either fluidized bed reactors^79^ or other reactor types.^80,81^ The conical spouted bed reactor meets the conditions to fulfill this goal, as its hydrodynamics avoids the defluidization of the bed caused by the agglomeration of the molten plastic.^82^ Furthermore, the short residence time of the volatiles inhibits the extent of the secondary reactions at the same time as it promotes the production of waxes (C_21+_) operating at low temperatures (80 and 92 wt % from HDPE and PP, respectively, at 450 °C)^68^ and the formation of light olefins at high temperatures (39 wt % from HDPE at 700 °C).^83^ Recently, an innovative cold plasma assisted pyrolysis reactor has been proposed to maximize the production of ethylene from HDPE.^84^

The study of fast pyrolysis of other non-polyolefinic polymers has also been focused on the recovery of monomers and raw materials. The pyrolysis of polystyrene (PS) in a conical spouted bed reactor allows attainment of 70.6 wt % yield of styrene at 500 °C, together with other commercially interesting fractions, such as fuels and aromatics (benzene and toluene).^85^ Poly(methyl methacrylate) (PMMA) is thermally degraded from 280 °C, and the yields of the monomer methyl methacrylate (MMA) and of ethyl acrylate (EA) reach values of 86.5 and 6.19 wt %, respectively, at 450 °C.^86^ Poly(ethylene terephthalate) (PET) is a very thermostable polymer, whose decomposition starts at 300 °C and reaches a significant degradation level above 400 °C. High temperatures increase the yield of gases and slightly that of liquid, whereas the yields of solid and residue are reduced.^87^ The main compound in the liquid product is acetaldehyde, reaching a yield of 11.1 wt % at 500 °C. In the solid product, in turn, benzoic acid is the main compound with a yield of 27.0 wt % at the same temperature.

#### 4.1.1 Yield and Composition of the Liquid Product and Interest in It as a Fuel

The main goal of many studies has been the production of a liquid product or PPO for its use as a fuel, directly or after being upgraded in refinery units. According to the number of carbon atoms in the molecules within the PPO, it is commonly divided into three different lumps: gasoline (C_5_–C_11_), diesel (C_12_–C_21_), and waxes (C_21+_). Within this context, Palos et al.^88^ have proven that the PPO obtained in the slow pyrolysis of HDPE at 430 °C has a composition similar to that of vacuum gas oil (VGO), which is the common feedstock of FCC units in refineries. Nevertheless, above 460 °C (independently of the reaction time), pyrolysis oil will have a distillation profile similar to that of light cycle oil (LCO), suitable to be fed on its own or cofed into a hydrotreatment unit.

The gasoline fraction obtained by Kumari and Kumar^89^ in the pyrolysis of HDPE has a suitable composition to be used as a motor fuel. Its low content of olefins and suitable content of aromatic compounds make up a stable gasoline fraction with good octane number. Dobó et al.^90^ studied the behavior of the gasoline fraction in the PPO obtained with different polymers (HDPE, LDPE, PP, PS) in an internal combustion engine. The fuel consumption was reduced with all the PPOs compared to that with a 95 research octane number (RON) gasoline. Indeed, this effect was maximized with the LDPE-derived gasoline fraction, reaching a reduction of 6.1–7.8 wt %. However, higher emissions were registered with the gasoline fraction obtained from the PPOs. Accordingly, based on the emissions obtained in the combustion of commercial gasoline, the fuels produced from HDPE, LDPE, and PP led to the highest emissions of CO, whereas NO_*x*_ emissions increased with the PS-derived gasoline.

Owusu et al.,^91^ in turn, focused their research on the diesel fraction. These researchers obtained their best results with HDPE and PP. However, the PPO obtained with PS requires an additional processing stage prior to be used in diesel engines. The performance in a diesel engine of different blends of commercial diesel and the PPO obtained from a mixture of plastics (mainly composed of styrene–butadiene and polyester) led to a longer ignition delay, higher cylinder peak pressure, and higher heat release rate caused by the lower cetane number of the blend.^92,93^ Furthermore, the engine thermal efficiency decreased by 3–4 wt % in comparison with that obtained with commercial diesel, and the emissions (including hydrocarbons, CO, and NO_*x*_) increased with the content of PPO in the blend. Based on the results reported by other authors, the effects on the thermal efficiency and on the emissions depend on the composition of the PPO and therefore on the type of plastic and operating conditions in the pyrolysis (especially on temperature). Thus, Das et al.^94^ reported that the suitable content of PPO in the blend with a commercial diesel is 20 wt %. Singh et al.^95^ determined that blends with contents of up to 50 wt % PPO allowed good performance with a slight decrease in the thermal efficiency compared to that obtained with the commercial diesel. Chintala et al.^96^ studied the performance and the emissions upon feeding PPO obtained from a mixture of waste plastics into a diesel engine at different brake mean effective pressures (BMEPs). The results showed that the thermal efficiency is comparable to that obtained with the commercial diesel. Furthermore, registered emissions (hydrocarbons, CO, and smoke) were also similar for low values of BMEP (1.8–3.8 bar), but higher emissions were obtained for high values of BMEP (5.8–10.8 bar). However, a notable reduction in the emissions of NO_*x*_ was observed at 10.8 bar because of the lower in-cylinder temperature. Gala et al.^97^ have compared the PONA (paraffins, olefins, naphthenes, and aromatics) analysis results (displayed in Table 3) of the PPO resulting from pyrolysis of industrial plastic waste (IPW), postconsumer colored (PCPW) and white plastic (PWPW) film waste in a pilot scale plant (80 kg h^–1^). As observed, the content of paraffins was higher in the postconsumer plastic films (50.5 and 57.8 wt % in PCPW and PWPW, respectively) than in the industrial one (38.7 wt %). Moreover, the PPOs complied with the hydrocarbons (50 vol %) in the diesel boiling point range (180–380 °C) and a blend of this fraction with the commercial diesel (50/50 vol %) met the requirements for being used in the EU as a fuel in diesel engine vehicles.

Arabiourrutia and co-workers^98^ characterized the waxes obtained in the fast pyrolysis of LDPE, HDPE, and PP by several techniques (gel permeation chromatography, simulated distillation analysis, and high heating value measurements). They divided their products into light and heavy waxes, establishing that overall, all the products obtained were suitable to be used as fuel. Moreover, they have observed that the results obtained with all the addition polymers were quite similar.

An interesting strategy that allows maximizing the selectivity toward certain products lies in the use of acid catalyst *in situ* in the pyrolysis reactor. Mark et al.^13^ have reviewed the different catalytic technologies used in the cracking of plastics and emphasized the role of the configuration and porous structure of catalyst particles in the yields and product distribution. The reaction mechanism in the catalytic pyrolysis (catalytic cracking) occurs through intermediate carbocations,^99^ at low temperatures and with a narrower product distribution. Furthermore, pyrolysis (thermal cracking) mechanism occurs with free radicals as intermediates, which induces random scission and chain-end scission reactions in the cracking of polyolefins.^100^ Pursuing the goal of maximizing the content of aromatics in the liquid product, Renzini et al.^101^ reached a selectivity of almost 100% with a Zn-impregnated ZSM-11 catalyst. Elordi et al.^102^ tested HZSM-5, HY, and H-Beta zeolite-based catalysts in the pyrolysis of HDPE. They observed that HZSM-5 zeolite promoted the formation of light olefins (yield of ca. 58 wt %), whereas HY and H-Beta zeolite-based catalysts allowed high yields (ca. 45 wt %) of nonaromatic C_5_–C_12_ hydrocarbons. Studying the effect of the acidity of the HZSM-5 zeolite demonstrated that slightly acidic catalysts with low acid strength promoted the yield both of light olefins (59.8 wt %) and of nonaromatic compounds, with the latter being similar to that of the gasoline fraction (32.1 wt %).^103^ In these works, emphasis has been placed on the relevance of the porous structure and acidity of the HZSM-5 zeolite for attenuating the deactivation caused by coke deposition. Using HZSM-5 zeolites, Wang et al.^104^ maximized the production of monocyclic aromatics in the pyrolysis of PC (polycarbonate) and PS.

Kassargy et al.^105^ extended the pyrolysis experiments to PE and PP using USY zeolites as catalysts. They obtained an average yield of 58.5 and 36 wt % of gasoline and diesel-like fuels, respectively. Elordi el al.^106^ used an equilibrated FCC catalyst (USY zeolite embedded in a macroporous structure) agglomerated with bentonite (50 wt %). This strategy pursued the aim of extending the life cycle of a refinery waste catalyst that could be obtained at very low price. Indeed, the yields of light olefin and gasoline fractions obtained (28 and 50 wt %, respectively) supported that catalytic pyrolysis of polyolefins could be integrated in a refinery. Additionally, Valanciene et al.^107^ also used an equilibrated FCC catalyst in the pyrolysis of waste industrial and automotive plastics, intensifying the formation of branched C_7_–C_9_ hydrocarbons.

Acid mesoporous catalysts have also been subjects of study. Thus, Li et al.^108^ compared different mesoporous materials (Kanemite-derived folded silica, Al-MCM41, and Al-SBA15) in the cracking of PE and PP. These catalysts lead to lower yields of gases and higher yields of aliphatic liquid products. Furthermore, Lee and Park^109^ have used commercial Al-MSU-F and desilicated β-zeolite for the catalytic pyrolysis of PE and PP. These authors observed that the properties of the catalysts strongly affect the results obtained, as higher yields of light and aromatic products are attained with the desilicated β-zeolite.

Carbonates have also been assessed as catalysts in the pyrolysis of polyolefins. MgCO_3_ was selected by Kunwar and co-workers for the pyrolysis of HDPE^81^ and of mixtures of PP, HDPE and medicine bottles.^110^ This catalyst leads to higher yields of diesel-range fraction. Singh et al.^111^ opted for testing CuCO_3_ for the pyrolysis of HDPE, obtaining high yields of liquid product (85–92 wt %). In another work, Singh^112^ performed the pyrolysis of virgin and waste HDPE using CoCO_3_ as catalyst, and they obtained slight differences in performance, with the liquid yields being very high (91 wt %).

With the aim of going a step further in this research topic, numerous authors have investigated the catalytic pyrolysis of postconsumer plastic mixtures.^81,113−119^ Among these works, the one by Sangpatch et al.^114^ is noteworthy. These researches have used local resources to synthesize the catalysts. Specifically, they used cogon grass, which is a native species in their region, as a source of silica for preparing silica–alumina catalysts. In the same line, Eze et al.^120^ have synthesized Y zeolites from kaolin extracted in their area. Based on the same concept of increasing the sustainability of the process, Li et al.^121^ have used the biochar produced in the pyrolysis of poplar woodchips as catalyst for the pyrolysis of LDPE and HDPE. They observed that the effect of biochar addition changed depending on the plastic used. Thus, biochar promoted the formation of gases (especially propane) in the pyrolysis of LDPE, while the formation of waxes was promoted in the pyrolysis of HDPE.

Another polyolefin pyrolysis strategy, in which monomer recovery is the aim, consists in using a tandem of two different setups connected in line. Artetxe et al.^122^ produced in the first stage (using a conical spouted bed reactor at 500 °C) a stream rich in waxes, which was thermally cracked in a second stage (at 850–900 °C). Final products were composed of 77.4 wt % light olefins, in which 40.4%, 19.5%, and 17.5% were ethylene, propylene, and butenes, respectively. The used HZSM-5 zeolite in the second stage causing various effects:^123^ (i) reduction in cracking temperature to 550 °C; (ii) decrease in the yield of olefins to 62 wt %; (iii) formation of aromatics within the gasoline fraction (C_5_–C_12_). The same strategy was used by Muhammad et al.^124^ in the catalytic pyrolysis of real and simulated mixtures of plastics. Even though the feeds used were different, these authors also observed a shift toward lighter products and the formation of 1-ring aromatics (benzene, toluene, xylenes, ethyl benzene, and styrene) when using HZSM-5 zeolite in the second stage. Akubo et al.,^125^ in turn, used Y zeolite-based catalysts loaded with Co, Ga, Fe, Mo, Ni, and Ru for cracking the volatiles of the pyrolysis of HDPE. The presence of metallic promoters led to the production of highly aromatic liquid products (between 97 and 99 wt %), at the expense of promoting the coke deposition on the catalysts.

#### 4.1.2 Kinetic Modeling

Most of the kinetic studies about the pyrolysis of plastics have been carried out by means of TG analysis, adjusting the mass loss results to an *n* order equation (commonly between 0.5 and 1) with activation energies and frequency factors within the 80–280 kJ mol^–1^ and 10^10^–10^18^ s^–1^ ranges, respectively.^126−131^ Aguado et al.^132^ determined that a conical spouted bed reactor is appropriate for kinetic studies. The rapid fusion of the plastics, high heat transmission velocity, and fast volatilization of the waxes obtained as pyrolysis primary products reduce the limitations of the thermogravimetric techniques.

**Figure 5:**
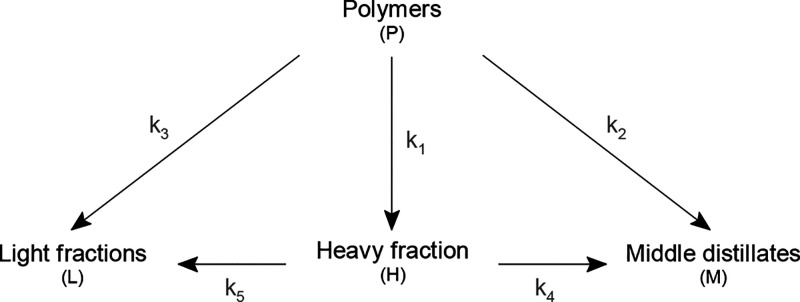
Kinetic scheme for the pyrolysis of polyolefins. Adapted from the work by Ding et al.^133^

**Figure 6:**
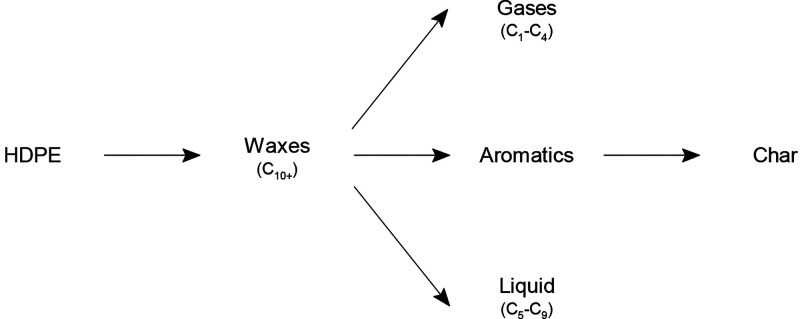
Kinetic scheme proposed for the thermal pyrolysis of HDPE. Adapted from the work by Aguado et al.^134^

Aiming at the production of fuels and raw materials, the kinetic models that group the different products in lumps are more attractive. Ding et al.^133^ computed the kinetic parameters of the pyrolysis of HDPE and mixed plastics (scheme shown in Figure 5). Considered lumps were light fraction (L), middle distillates (M), and heavy fraction (H). Aguado et al.^134^ used Principal Component Analysis methodology to establish the reaction scheme in Figure 6 for the pyrolysis of HDPE. Furthermore, various works have revealed that the presence of zeolite-based catalysts reduces the activation energy of the pyrolysis of plastics reducing, at the same time, the required temperature.^135−137^ Indeed, the acidity and shape selectivity of the zeolite are key factors for controlling product distribution and for attenuating the deactivation caused by coke deposition.^138^ The magnitude of the effects of using acid catalysts on the product distribution has been quantified by lump kinetic models.^139,140^ In this context, Till et al.^141^ have established a kinetic model composed of 6 lumps and 10 individual kinetic steps to describe the pyrolysis of a HDPE/PP/LDPE mixture.

### 4.2 EOL Tires

#### 4.2.1 Product Distribution

**Figure 7:**
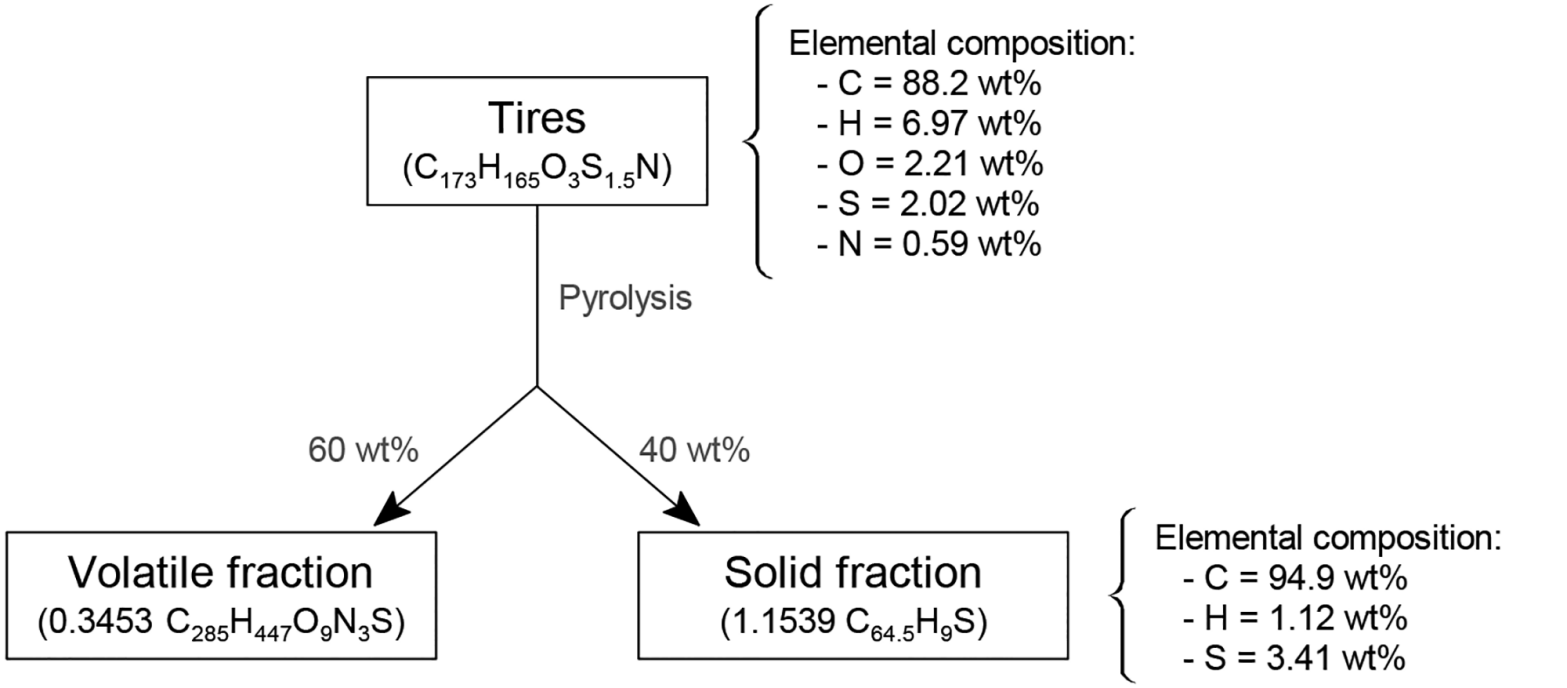
Product distribution in the pyrolysis of tires. Adapted from the work by Martínez et al.^16^

The pyrolysis of EOL tires is considered as a promising route for the valorization of this solid waste, as the products streams (gas, char, and TPO) are of high heat value.^142^ Martínez et al.^16^ described the pyrolysis of tires considering the average composition of the products (Figure 7), distinguishing a volatile fraction, which is mainly composed of tire pyrolysis oil (TPO), from a solid fraction (40 wt %), which is basically adulterated carbon black (CBp).

However, the composition and the yields of the different product fractions are strongly affected by the operating conditions, tire formulation, reactor type, and the presence of a catalyst. The most interesting reactor types for the continuous pyrolysis of EOL tires are moving-bed, rotatory kiln, fluidized bed, and conical spouted bed reactors.^71^ The aim of the noncatalytic pyrolysis is the production of the liquid product (TPO) for its use as fuel, even though the economic viability of the process requires the valorization of the CBp and the separation of high-value added chemicals from the TPO (isoprene, d-limonene, *o*-cymene, *o*-xylene, toluene, and ethyl-benzene).^21^ The pyrolysis of EOL tires has a low environmental impact, as the presence of metals in the TPO and in the char is rather low. However, both the TPO and the gas fraction require a subsequent desulfurization stage.^19^

Lopez et al.^143^ pyrolyzed two different types of tire materials with different contents of natural rubber and synthetic polymers (polystyrene and polybutadiene, respectively) in a conical spouted bed reactor. They observed that product distribution was barely affected by tire formulation, but strongly influenced product composition. The higher the content of synthetic rubber in the tire formulation, the higher the yields of benzene, toluene, and xylenes (BTX). The yield of limonene followed the same trend; that is, the maximum yield was obtained in the pyrolysis of synthetic rubber. Similar results were obtained by Singh et al.^144,145^ in the pyrolysis of different automotive waste tires and by Tang and co-workers^146^ in the pyrolysis of waste rubber and polyurethane bicycle tires.

Pyrolysis under vacuum solves one of the important limitations for the scale up of the process, as the N_2_ flow required for operating under fluidized or spouted bed regimes is reduced. In the case of the conical spouted bed reactor, operation at 0.25 atm in the 400–600 °C range required a N_2_ flow rate 3.5 times lower than that required under atmospheric pressure.^147^ Vacuum has a marked effect on product distribution,^75^ increasing the yield of the liquid fraction and promoting the formation of less CBp, with average surfaces above 90 m^2^ g^–1^. Moreover, the yield of isoprene was increased, to the detriment of the yield of limonene, because vacuum attenuates the dimerization reaction of isoprene to limonene.

The catalytic pyrolysis of waste tires has also been approached in the literature in order to improve product distribution.^24^ Arabiourrutia et al.^148^ and Olazar et al.^149^ studied the *in situ* catalytic pyrolysis of EOL tires using HZSM-5, HY, and H-Beta zeolites. The HZSM-5 zeolite promoted the formation of gases (increasing the yield of propylene and butadiene over that obtained without catalyst) and of hydrocarbons within the gasoline fraction, with an average content of 20 wt % of BTX aromatics. Conversely, the HY zeolite produced a heavier liquid product, with hydrocarbons prevailing within the diesel fraction. However, in both cases, the HHV of the TPO was lower than that obtained in the thermal pyrolysis.

Williams and Brindle studied the catalytic cracking of the volatiles obtained in the pyrolysis of tires using acid zeolite catalysts (HZSM-5 and HY zeolites).^150,151^ Based on their tandem strategy, these authors observed that the catalytic treatment reduced the yield of the liquid fraction, which was converted into gas and coke. In spite of this, the concentration of BTX aromatics was significantly increased with the catalysts, especially with the HY zeolite.

Although the pyrolysis of tires aims for the production of the TPO, yield of which reaches values of 58.2 wt % at 475 °C in a conical spouted bed reactor,^69^ various byproducts are also obtained. Among them, CBp (with an average yield of 35 wt %) is the most interesting one as its surface area and structure are similar to those of commercial CB.^152^ Therefore, once it has been subjected to atomization dispersion and high temperature sputtering drying, it can be used for the preparation of rubber composites.^153^ Moreover, the low ash content (<10 wt %) and high volatile matter (>70 wt %) make it appropriate for use as adsorbents in pollution control and as biochar for soil amendment.^22,154^

#### 4.2.2 Composition and Properties of the Liquid Product as a Fuel

TPO is a brownish liquid with the appearance and smell of petroleum fractions. It is a complex mixture of organic compounds of 5–24 carbon atoms, with a H/C molar ratio of ∼1.4 and a large proportion of aromatics. Its aromaticity and, especially, the content of polyaromatic hydrocarbons (PAHs) (naphthalene, phenanthrene, fluorene, biphenyls, etc.) increases with temperature because aliphatic cyclization reactions are enhanced, together with the combination reactions involving aliphatics and aromatic free radicals.^155,156^

Additionally, TPO can be the source of various raw materials, such as dl-limonene, dipentene, and isoprene. The concentration of these chemicals in TPO is strongly affected by pyrolysis conditions, especially temperature and heating rate.^157−159^ In a conical spouted bed reactor, the concentration of dl-limonene may be as high as 26.8 wt %,^160^ with the concentration of PAHs being low (2.42 wt %), which is a consequence of the short residence time of the volatiles. Moreover, the TPO obtained in this reactor type is very light, as more than 60 wt % of the compounds are within the gasoline fraction (C_5_–C_12_), with a notable concentration of BTX. In addition to isoprene, most of the compounds in the C_5_ fraction are olefins. The C_6_ and C_7_ fractions are mainly composed of diolefins, whose yield increases with temperature. The most representative compound in the C_8_ fraction is styrene, whereas in the C_9_ fraction, they are indene and benzene derivatives. Finally, 1-methyl-4-(1-methylethyl)benzene and 1-methyl-2-(1-methylethyl)benzene are found in the C_10_ fraction, which are formed by the dehydrogenation of limonene. Furthermore, the heaviest fraction of the gasoline (C_11_–C_12_) contains aromatic compounds, which are benzene, naphthalene, and indene derivatives, as well paraffinic and olefinic compounds.^161^

Table 4 shows the average fuel properties of TPOs obtained under different conditions, which have been reported by Rowhani and Rainey.^14^ These authors have compared the average TPO features with those of commercial gasoline and diesel. The similarity between the properties of the TPOs and the commercial diesel has promoted the testing of TPO as an alternative fuel by feeding either on its own or blended with diesel. İlkılıç and Aydin^162^ tested the behavior of a direct injection engine under different blending ratios of TPO and diesel. These researchers concluded that the engine operated efficiently and without requiring any modification with TPO contents of up to 35 wt %. However, blends with TPO content of ∼50 wt % led to a considerable increase in particulate matter, CO, SO_2_, and smoke emissions. Similar results were obtained by Murugan et al.^163^ blending the TPO in ratios of up to 50 wt % with automotive diesel. Other authors^164,165^ established that the feeding of only TPO into a standard rail diesel engine requires tailored injection strategies for optimum behavior of the engine.

With the aim of improving the ignition of the TPO/diesel blend, Wang et al.^166^ proved that an increase in the pyrolysis temperature of the tires entailed an increase in the HHV (to the detriment of the yield of TPO). Consequently, the volume of the blend required for ignition was reduced. Additionally, Hariharan et al.^167^ investigated the effect of adding diethyl ether and concluded that this addition led to a reduction in the emissions of NO_*x*_. However, given the high content of aromatic compounds and low H/C ratio, the emissions of unburned hydrocarbons and CO were 38% higher than in the case of the conventional diesel.

Nevertheless, some physicochemical properties of the TPO,^71,168^ such as low cetane index (∼40), high viscosity (∼6.3 cSt), high content of aromatics (∼65 wt %), and total content of sulfur (∼14000 ppm), are serious disadvantages for its direct use as fuel in internal combustion engines. Depending on the technology used for its production, TPO may contain sand or coal particles or alkali metals that could damage parts of the engine.^169^ Furthermore, it generates a higher amount of coke in the injectors and higher emissions of unburned hydrocarbons, particulate matter, SO*_x_*, and NO*_x_*.^170^ Its direct use in internal combustion engines may delay the ignition of the engine requiring a bigger volume of oil in the cylinders to start ignition. This, in turn, entails pressure increase within the cylinders and therefore a decrease in the engine performance.

Alvarez et al.^161^ made a detailed analysis of the TPO obtained in a conical spouted bed reactor in the 425–475 °C range. The simulated distillation analysis showed that approximately 70 wt % corresponds to the diesel range. These authors pointed out the need for reducing the content of sulfur, nitrogen, and aromatics for use as fuel. The content of sulfur (up to 1.6 wt %) appears in the form of benzothiazol, dibenzothiophene, and its alkylated derivatives.^171^ Within this context, some strategies have been tested to obtain a sulfur free liquid product in the pyrolysis stage. They have consisted in using *in situ* catalysts of CaO, MgCl_2_, or NaOH, but moderate reductions have been obtained, as the maximum sulfur reduction has only accounted for 35 wt %.^162,172,173^ Hence, the required sulfur reduction must be carried out by means of a desulfurization or mild hydrotreatment process in order to use TPO as fuel. Furthermore, TPO can be fractionated to obtain different quality fuels,^174^ and the heaviest fractions can be used as plastifiers in different rubber formulations or as a substitute for asphalt concrete.^175^

#### 4.2.3 Kinetic Modeling

Conventionally, the EOL tire pyrolysis kinetics has been determined by thermogravimetric means quantifying the evolution of mass loss with time.^176−270^ The differences between the kinetic parameters, that is, pre-exponential factor, activation energy, and reaction order, are a consequence of the mass and energy transfer limitations during the experiments and of the different composition of the EOL tires used in each work. The use of a fast heating microreactor allowed Aguado et al.^178^ to obtain kinetic data at higher temperature (within the 500–550 °C range) and under conditions similar to those of a continuous large-scale reactor. Olazar et al.^181^ used a conical spouted bed reactor to study the kinetics of the pyrolysis of EOL tires because of its isothermicity and high mass and energy transfer velocities between the phases. Some authors considered the heterogeneity of the EOL tires by means of kinetic models that evaluate the independent decomposition of their main compounds. Lopez et al.^147^ identified by DTG analysis the kinetics for the pyrolysis of individual components of the EOL tires. Those components were volatile components, natural rubber, and styrene–butadiene rubber, and their activation energies computed under vacuum (0.25 atm) were 43.5, 104.7, and 243.0 kJ mol^–1^, respectively. Lah et al.^183^ established a kinetic model that identified the kinetics of five different components of the EOL tires: (i) fabric materials, which include rayon, nylon, and aramid; (ii) wire; (iii) natural rubber; (iv) styrene–butadiene rubber; (v) butadiene rubber, together with the heat of reaction and the internal and external mass and heat transport phenomena.

**Figure 8:**
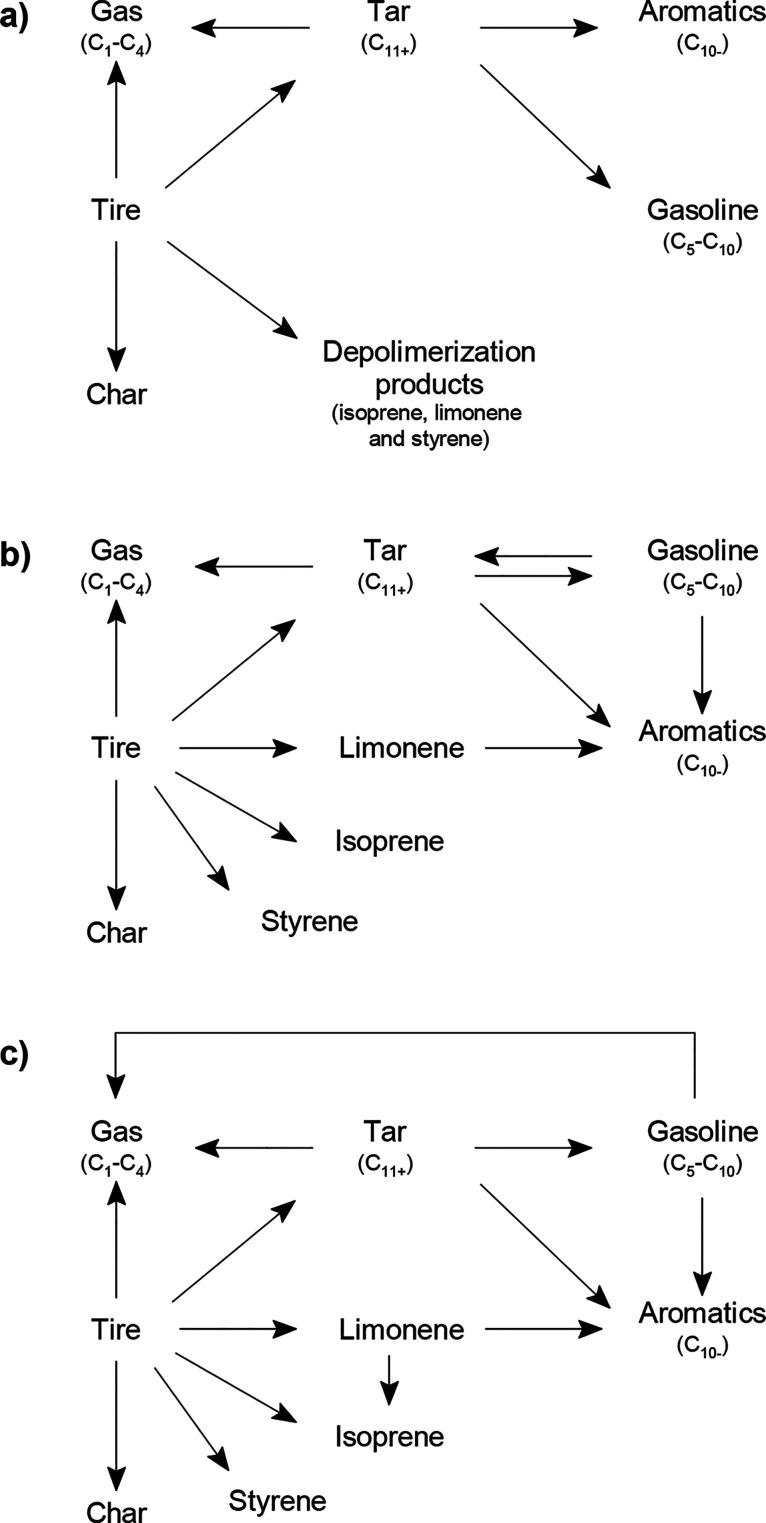
Kinetic scheme for the (a) thermal and (b, c) catalytic pyrolysis of tires with (b) HY and (c) HZSM-5 zeolites. Adapted from the work by Aguado et al.^189^

Pursuing the production of fuels and raw materials, the kinetic models that quantify the products distribution are of special interest.^184−188^ Aguado et al.^189^ used the Principal Component Analysis methodology for grouping the products into different lumps (Figure 8a): gas (CH_4_, C_2_–C_4_); monoaromatics C_10–_; nonaromatic gasoline (C_5_–C_10_); tar (C_11+_); char (CBp). The presence of acid catalysts has a strong influence on the kinetic scheme. The HY zeolite promotes the condensation and alkylation reactions that lead to the formation of aliphatic hydrocarbons in the gasoline fraction and of mono- and polyaromatics in the tar (Figure 8b). In contrast, the HZSM-5 zeolite fosters different cracking stages: (i) tar to monoaromatics and gases; (ii) limonene to the monomer isoprene; (iii) C_5_–C_10_ aliphatics to gases (Figure 8c).

## 5 Catalytic Cracking

### 5.1 FCC Unit

FCC units, which are available in most of the petroleum refineries worldwide, are used to produce high octane gasoline and light olefins from heavy streams obtained in the distillation of crude oil. They are composed of four sections:^190^ (i) the pneumatic transport reactor (riser); (ii) the stripper; (iii) the gas–solid separator; (iv) the regenerator. The process starts when the preheated feedstock, commonly vacuum gas oil (VGO) with a boiling point above 344 °C, is steam-atomized at 350–425 °C. Afterward, the atomized feedstock is mixed at the base of the riser reactor with the catalyst stream that comes from the regenerator at 650–700 °C. Note that, based on the different temperatures of the feedstock and the catalyst, the mixture ends with an average temperature of 530 °C. The steam-atomized feedstock sweeps the catalyst throughout the riser, which has a length of 25–40 m and a diameter of 0.6–1.2 m. Because of the cracking reactions, the gas stream expands, reaching velocities of 5–15 m s^–1^. The flow regime corresponds to a dense-phase pneumatic conveying system due to the high catalyst to oil ratio (4–9 g_cat_ g_feed_^–1^) and residence time of the gas and catalyst (3–8 s).

In the upper part of the reactor, cracking reactions reach their end, but in order to avoid undesired secondary reactions, the catalyst is separated from the products by high efficiency (99.995%) cyclones. Products exit through the reactor head and go to fractionation and concentration systems, with the average fractions being commonly as follows: dry gases (C_1_–C_2_) 3–5 wt %; liquefied petroleum gases (LPG, C_3_–C_4_) 8–20 wt %; gasoline (C_5_–C_12_) 36–60 wt %; light cycle oil (LCO, C_13_–C_21_) 12–20 wt %; heavy cycle oil (HCO, C_21+_) 10–15 wt %; coke 3–8 wt %.^190^ The deactivated catalyst goes to the stripping section, where interstitial and adsorbed hydrocarbons are removed from the catalyst by a counter current stream of steam (2.5 kg of steam per ton of catalyst). Once they have been separated, the hydrocarbons go to a fractionation column, whereas the catalyst goes to the bubbling-bed regenerator (10–15 m in diameter).

The catalyst inventory of an average FCC unit, which treats ca. 50 000 barrels per day (bpd), is 270–300 tons. FCC units perform between 100 and 400 cycles per day, and in each cycle, the catalyst spends most of the time in the regenerator (6–11 min), and only 3–8 s in the riser reactor. The content of coke at the entrance of the regenerator is 0.4–2.5 wt %, and it is removed by combustion at a temperature of 620–745 °C with an air velocity of 0.6–1.2 m s^–1^. This way, the catalyst is reactivated and acquires the sensitive heat required to satisfy the thermal requirements of the unit.^191^ Furthermore, the combustion gases that leave the regenerator drag the particles produced by the attrition phenomenon, and they must be retained and replaced by a stream of fresh catalyst.

USY zeolite has a well-defined crystalline structure with a cubic unit cell of 24.5–24.75 Å and a silica/alumina ratio between 3 and 6. The internal cavity of its super cage is 12 Å in diameter, with entries of 7.4 Å (12 oxygen atom rings). Each cavity is connected to the other four cavities, which are, in turn, connected to another four leading to the formation of the characteristic tridimensional structure of the Y zeolite. The crystals of the USY zeolite are embedded in a matrix commonly composed of alumina, silica–alumina and clay. The matrix plays a key role in the behavior of the catalyst within the reactor due to the following aspects: (i) it confers appropriate fluid dynamical properties, mechanical resistance, and thermal conductivity upon the catalyst particles; (ii) the macropores of the matrix (average diameter of 100–600 Å) ease heat and mass transfer and allow the diffusion of the heavy components of the feedstock; (iii) its acidic properties contribute to the cracking of heavy compounds; (iv) it retains poisoning substances (N and S containing molecules and metals, specifically Na) extending the life cycle of the catalyst and reducing their content in the products; (v) the coke formed inside the zeolite crystals flows outward and settles on the matrix attenuating the blockage of the zeolite channels. The main modifications of the USY zeolites lie in the steam ultrastabilization and ion-exchange with rare earth oxides (REO) to attenuate dealuminization and improve the hydrothermal stability required in the regeneration step.

**Figure 9:**
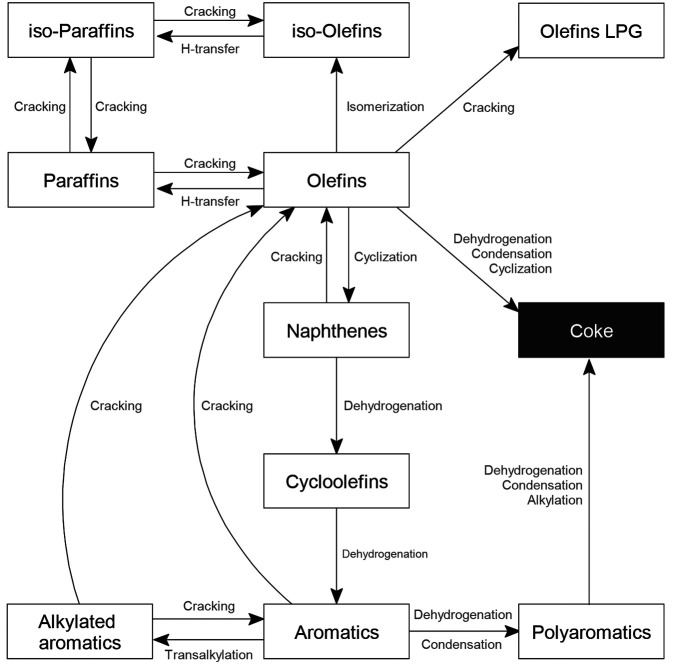
Main reactions occurring in the riser reactor of the FCC unit.

Figure 9 shows a scheme of the main reactions in which each type or family of hydrocarbons is involved in the catalytic cracking in the FCC unit.

### 5.2 Cracking of Waste Plastics

The results obtained under experimental conditions similar to those in the industrial FCC unit are detailed in this section. For the cracking of waste plastics, different strategies have been studied for their feed into the unit, such as (i) dissolution in conventional refinery streams and (ii) conversion into PPO, which is fed on its own or blended with the standard FCC unit feedstock.

#### 5.2.1 Plastics Dissolved in Conventional Refinery Streams

The direct cofeeding of plastics dissolved in conventional refinery streams (vacuum gasoil, VGO) has the advantage of not requiring additional pyrolysis facilities. However, this direct strategy shows several drawbacks: (i) a rigorous separation of polyolefinic plastics must be carried out in municipal solid waste collection and segregation points; (ii) plastics must be transported to the refineries, which is not an easy task given their low density; (iii) plastics must be dissolved in refinery streams. In short, a non-normalized and difficult to obtain feedstock would have to be handled in the refinery.

The first reference in the literature about catalytic cracking in a fixed bed MAT type reactor by feeding VGO blended with HDPE (5 and 10 wt %) at 510 °C reports a substantial production of gasoline from the HDPE plastic contained in the feed (10 wt %).^192^ Later, the cracking of polyolefins and polyaromatics under conditions similar to those of the industrial unit was studied on a riser simulator reactor with different types of catalysts: (i) equilibrated commercial FCC catalysts;^193,194^ (ii) commercial fresh catalysts and other in-house synthesized HY zeolite-based catalysts with different porous structures and acidities;^195,196^ (iii) catalysts prepared in the laboratory using HZSM-5 zeolites as additives.^195^ The solvents used in these studies for dissolving the plastic were VGO, which is the current FCC unit feed, and light cycle oil (LCO), which is a product stream of the FCC unit with a high content of aromatics.

The cofeeding of polyolefins with LCO increased the yield of gasoline and reduced that of coke. The content of aromatics was reduced in the gasoline fraction, at the same time as the contents of isoparaffins and olefins was increased, thereby leading to an increase in the quality of the gasoline fraction obtained. Moreover, the RON increased with temperature from 98.1 to 99.0 when 10 wt % PE was in the feed.^193^ The results obtained by cofeeding PP were quite similar. Furthermore, the use of HZSM-5 zeolite as an additive of the catalyst significantly affected product distribution. A notable increase in the yield of olefins was obtained, whereas the yields of aromatics, paraffins, and coke were reduced.^195^ These results were later on ratified by Marcilla et al.^197^ in a sand fluidized bed reactor and by Odjo et al.^198,199^ in a FCC pilot plant. Therefore, the viability of cofeeding polyolefins with VGO without affecting the yields and quality of the product streams is evident.

When 10 wt % PS was cofed with LCO, the conversion surpassed that obtained with pure LCO, the yield of gasoline increased to the detriment of that of dry gases, and the fraction of LPG was mostly olefinic, with propylene and isobutene being the main compounds. Additionally, it should be highlighted that 50 wt % of the styrene in the PS was recovered. The RON of the gasoline, between 97.2 and 95.4, was lower than that obtained in the cracking of pure LCO. This drop is a consequence of the lower content of isoparaffins and olefins. The results obtained by cofeeding PS-BD were qualitatively similar, even though the yield of the gasoline fraction obtained was 2 wt % lower.^193^

**Figure 10:**
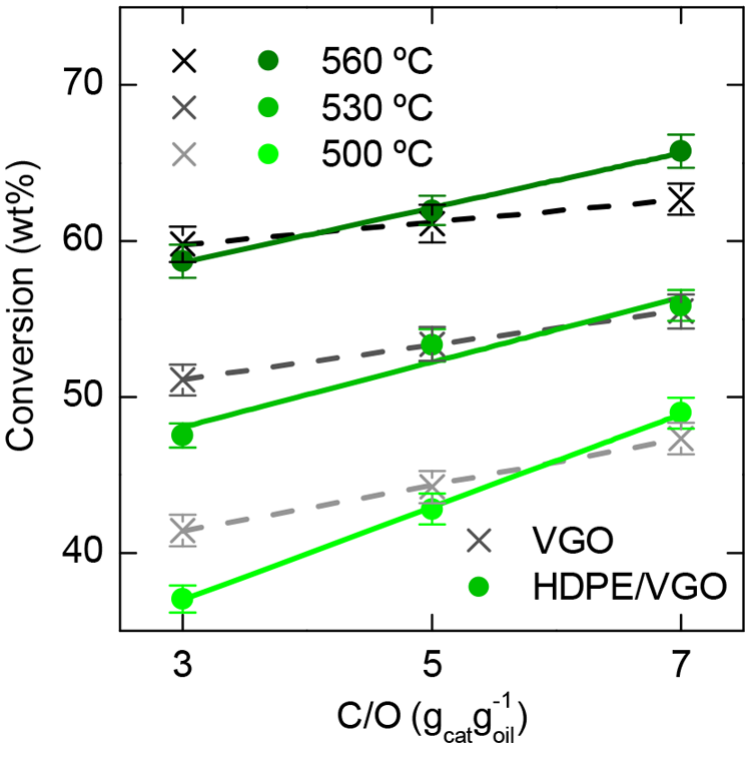
Evolution of conversion with C/O ratio in the cracking of the HDPE/VGO blend (solid lines) and raw VGO (dashed lines) at different temperatures.^200^

The conversions obtained in the cracking of a HDPE/VGO blend (10 wt % of HDPE) are compared in Figure 10 with those obtained in the cracking of pure VGO. The conversion attained with the blend was in the 37–66 wt % range, whereas with the VGO it was in the 41.4–62.7 wt % range. An increase in the catalyst to feed (C/O) ratio and, especially in temperature, allowed reaching higher conversions in the cracking of the blend.^200^ For low conversion values, that is low temperature and low C/O ratio, the reactivity of the blend was lower than that of the VGO. However, for a conversion of 58 wt %, the previously mentioned differences disappeared and the crackability of both feeds was similar. Under these conditions, the cofeeding of HDPE promoted the formation of LPG and gasoline fractions, to the detriment of dry gas and coke. The lower formation of coke led to a minor deactivation of the catalyst, which explained the higher yields of LPG and gasoline as well as the lower overcracking observed.^200^ This work also compared the composition of the gasoline fraction obtained in the cracking of the HDPE/VGO blend with that obtained in the cracking of the VGO, and several differences were observed in the concentration of all the families of hydrocarbons. Nonetheless, the operating conditions lead to similar trends for both feeds. Thus, higher temperatures involve higher concentrations of olefins and lower of the remaining families in the gasoline fraction. Furthermore, these researchers observed subtle differences in the impact of temperature. High temperatures reduced the difference between the concentration of linear and branched paraffins, and the concentration of aromatics reached its maximum value at 530 °C. The effect of increasing the C/O ratio lies in increasing the concentrations of *n*-paraffins and isoparaffins (especially at 500 °C) in the gasoline fraction and in reducing the concentration of olefins. These modifications in the composition of the gasoline fraction were a consequence of the promotion of hydrogen-transfer reactions. Similarly, the promotion of Diels–Alder reactions led to an increase in the concentration of aromatics in the cracking of the blend. However, the concentration of aromatics increased to a minor extent in the cracking of VGO, because fewer olefins were in this feedstock.

#### 5.2.2 Plastic Pyrolysis Oil (Waxes)

A previous step of pyrolysis of waste plastics would make much easier their valorization in refinery units. Plastics could be locally converted into liquid or waxy hydrocarbons in small pyrolysis units located near the municipal solid waste collection and sorting points. Accordingly, the subsequent transport of pyrolysis derivatives to the refinery would be easier as a small fleet of tanker trucks would be sufficient to collect all the products of medium-large geographical areas. Furthermore, this feed could be stored and mixed in the refinery oil terminals in order to attain a standard formulation prior to their treatment in the corresponding units.

Iribarren et al.^201^ determined by life cycle analysis that the combined strategy of pyrolysis and catalytic treatment is the most sustainable management strategy when the perspectives involving energy and environment are considered. Based on these positive points, various authors have approached the catalytic cracking of plastic pyrolysis waxes, either neat^202,203^ or dissolved.^204,205^

Rodríguez et al. studied the catalytic cracking of neat HDPE pyrolysis waxes in two different works. First,^202^ these authors studied the suitability of the FCC unit for the production of fuels from the HDPE pyrolysis waxes. Accordingly, they performed a parametric study where temperature and catalyst to oil ratio were investigated. Moreover, the results were compared with those obtained in the cracking of VGO in order to analyze their trends. Overall, HDPE pyrolysis waxes were less reactive than VGO. Temperatures above 550 °C and C/O ratios of 7 g_cat_ g_feed_^–1^ were required to obtain higher conversions with the waxes. The composition of the gasoline fraction was also different, as it depended on the composition of the feedstock. Thus, the gasoline fraction was more paraffinic and olefinic and less aromatic than that obtained from the VGO. The same authors tested different FCC equilibrated catalysts in the cracking of the waxes^203^ and concluded that the properties of the catalyst played a significant role in product distribution. In fact, catalysts with low acidity promoted the formation of gasoline with low content of aromatics, suitable to be marketed after a mild hydrotreatment stage, whereas highly acid catalysts were appropriate for the production of commodities, such as C_5_ and C_6_ olefins.

**Figure 11:**
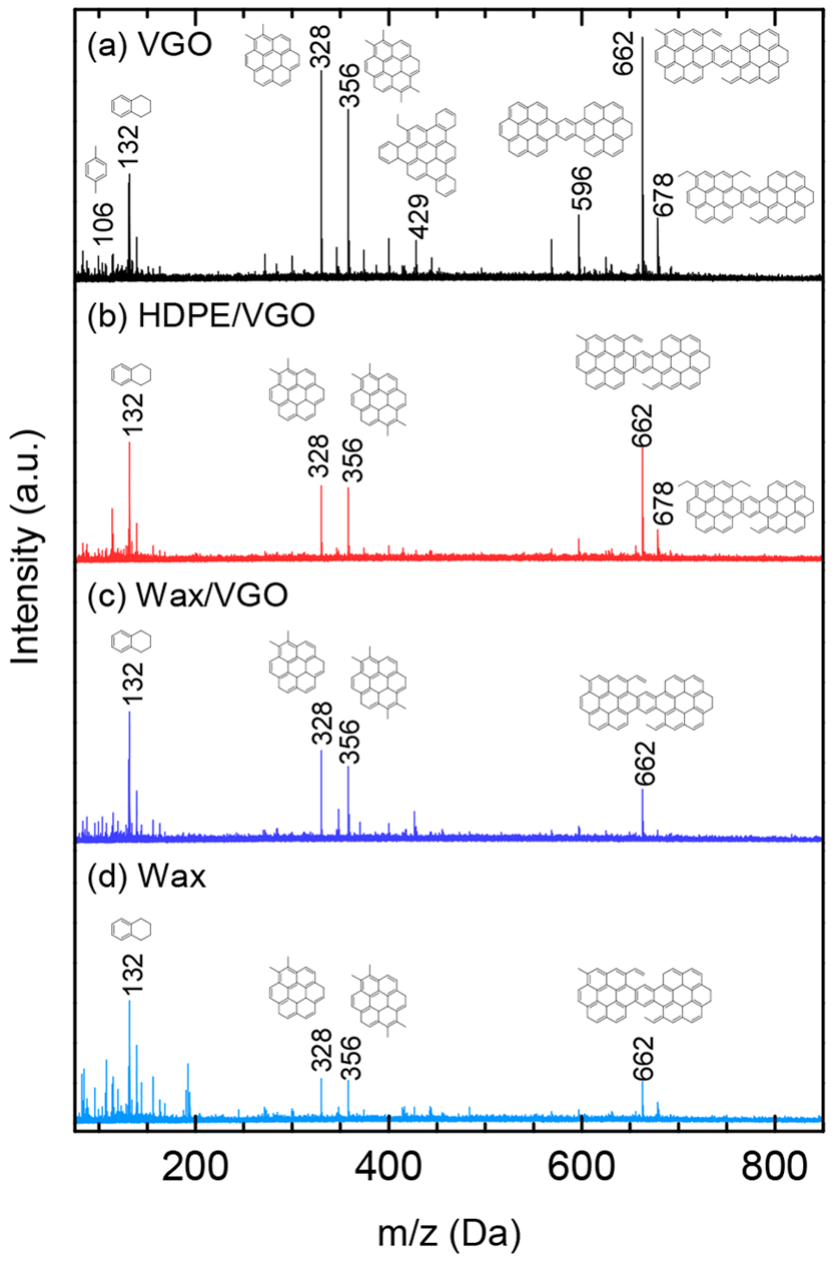
LDI TOF-MS spectra and main coke species detected in the spent catalyst used in the catalytic cracking of VGO, HDPE/VGO blend, HDPE wax/VGO blend, and neat HDPE wax. Adapted from the work by Rodríguez et al.^206^

Nonetheless, the cofeeding of waste plastic pyrolysis waxes with a benchmark feed provides a more realistic approach concerning the integration of waste plastic valorization into refineries. This strategy was first approached by Lovás et al.,^204^ as they studied the cocracking of HDPE and PP pyrolysis waxes blended with atmospheric gas oil and hydrotreated gas oil in a MAT experimental apparatus. They concluded that both blends (with HDPE and PP) improved the crackability of the hydrotreated gas oil. Moreover, they observed that the cofeeding of HDPE promoted the formation of light olefins, whereas that of PP increased the formation of the gasoline fraction. Afterward, Rodríguez et al.^205^ investigated the cocracking of HDPE pyrolysis waxes and VGO in a riser simulator reactor. They determined that the cofeeding of the HDPE pyrolysis waxes had remarkable effects on the process. Thus, the cofeeding inhibited the secondary cracking reactions, which promoted the formation of the dry gas fraction, and increased the yields of LPG and gasoline fractions. Moreover, a reduction in the content of coke was observed because of the higher H/C ratio of the blend. Overall, higher contents of olefins and paraffins and lower contents of aromatics were obtained. Consequently, a LPG fraction rich in ethylene, propylene, and butylenes was obtained with the blend. In addition, a higher quality gasoline fraction was obtained, with values of the octane index being about 103. With regard to coke deposition,^206^ the cofeeding of HDPE pyrolysis waxes significantly lessened the formation of coke on the catalyst. In addition, its nature was rather different from that obtained in the cracking of neat VGO (Figure 11). Hence, it was less aromatic and more aliphatic and it contained long olefinic chains, which made regeneration easier and could extend the life cycle of the catalyst.

### 5.3 Cracking of Tire Pyrolysis Oil

Even though the pyrolysis of scrap tires has been extensively analyzed in the literature (section 4.2), the catalytic cracking of TPO has been barely studied. A comparison of the properties of the TPO with those of the streams currently used in the refinery for the production of fuels (Table 5) shows that the TPO could be potentially cofed with these feeds.

**Table 5:** Properties of the TPO, LCO, and VGO

	TPO					
properties	stirred tank	rotary kiln	fixed bed	CSBR	LCO	VGO
density (kg L^–1^)	0.91	0.96	0.83	0.89	0.78	0.89
viscosity 40 °C (cSt)	6.30				3.30	21.0
HHV (MJ kg^–1^)	42.0	41.7	42.7	44.0	44.8	46.0
flash point (°C)	20	17	65		79	75
carbon residue (wt %)	2.20	1.78				<0.35
elemental analysis						
C (wt %)	88.0	84.3	79.6	87.2	85.5	87.1
H (wt %)	9.40	10.4	10.0	10.6	12.4	12.8
N (wt %)	0.45	0.42	0.94	0.45	0.15	0.05
S (wt %)	1.50	1.54	0.11	1.22	1.40	0.90
proximate analysis						
ash content (wt %)					0.01	0.02
moisture (wt %)	4.60	0.88			0.05	0.10
simulated distillation						
IBP (°C)	100		38.5	129	139	218
90% BP (°C)	355			455	352	507

Adapted from the work by Hita et al.^71^

Rodríguez et al. have studied the cracking of pure TPO obtained in a conical spouted bed reactor,^207,208^ the cracking of TPO dissolved in VGO,^209^ and the nature and location of the coke formed in this process.^210^ Initially,^208^ these researchers studied the effect of operating conditions, that is, temperature, C/O ratio, and contact time. Accordingly, they used a riser simulator reactor and an equilibrated FCC catalyst in order to perform the testing at industrial conditions. These authors observed that high temperatures promoted cracking reactions leading to the formation of light compounds within LPG and gasoline fractions. Moreover, they also verified that olefin cyclization reactions and C–C bond cracking reactions from aromatics were boosted at high temperatures, while hydrogen-transfer reactions were inhibited. Consequently, the content of olefins increased in the dry gas and LPG fractions and the content of aromatics and paraffins in the gasoline fraction. Furthermore, higher values of C/O ratios and longer contact times boosted cracking, hydrogen-transfer, and condensation reactions, promoting the paraffinicity and aromaticity of the reaction products. Later, they assessed the effects that catalyst properties have on the conversion, distribution, and composition of the reaction products.^207^ Three different equilibrated FCC catalysts supplied by industrial providers were tested in the work. They concluded that the properties of the catalyst are highly influential. Thus, high total acidity and acid strength of the catalyst promoted the extent of the cracking reactions. Moreover, the textural properties of the matrix (meso- and macropores of the catalyst) play a significant role in the diffusivity of the bulky molecules.

**Figure 12:**
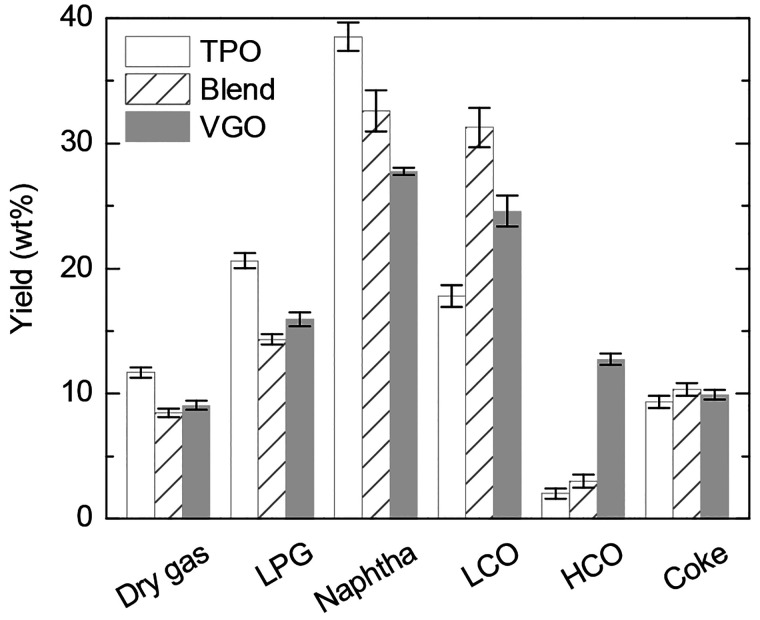
Comparison of the yields of product lumps obtained in the cracking of TPO, VGO, and the blend of TPO/VGO with 20 wt % TPO. Adapted from the work by Rodríguez et al.^209^

Afterward, Rodríguez et al.^209^ tried a more realistic approach, as they studied the cocracking of TPO with the conventional FCC unit feedstock, VGO. Furthermore, they compared the results obtained with the TPO/VGO blend with those obtained in the cracking of the pure feeds separately. Figure 12 shows the product yield distribution obtained with the pure feeds and the blend. As observed, there are various synergistic effects when the blend is fed. Thus, the addition of 20 wt % TPO to the blend promoted the cracking of the HCO fraction, as its extent is closer to that obtained with pure TPO than with pure VGO. Furthermore, overcracking reactions that commonly lead to the formation of gas products were inhibited, as the lowest yields of dry gas and LPG fractions were obtained with the blend. Consequently, the blending promoted the formation of naphtha and LCO fractions, improving the results obtained for the VGO.

The formation of coke is of crucial relevance in the reaction extent and regeneration of the catalyst. By means of several analyses, the nature and location of the coke deposited in the cracking of the TPO has been determined,^210^ demonstrating that the coke derived from TPO was lighter and more aliphatic than that of the VGO and located on the outside of the micropores. Conversely, the coke is commonly located within the micropores of the catalyst in the cracking of VGO. Therefore, the addition of TPO to the cracking process reduced the deactivation of the catalyst.

## 6 Hydroprocessing

Hydroprocessing units are commonly available in refineries for low severity or mild hydrotreatment with the aim of removing heteroatoms from the feeds. Afterward, these streams can either be sent to another unit or be marketed as fuels. Nevertheless, the presence of hydrocracking units (capable of reducing drastically the presence of aromatics and generating linear hydrocarbon chains) is not so common, and they can only be found in innovative refineries.^211,212^ The availability of these hydrocracking units is a key factor to face the increasingly restrictive environmental rules and the possible inclusion of new feeds (bio-oil or waste derivatives, such as those of plastics and EOL tires).

### 6.1 Hydroprocessing Units

Hydroprocessing is a refinery stage in which petroleum-derived oils are upgraded under high pressures of H_2_ and high temperature. It aims toward the adaptation of liquid fuels to environmental requirements by means of (i) the hydrogenation of the unsaturated compounds, especially aromatics, (ii) the removal of impurities (N, S, O, and metals), and (iii) the cracking of heavy compounds improving the yields of gasoline and diesel fractions.^213,214^ This process is carried out under a broad range of conditions, and therefore hydroprocessing units are denoted as (i) hydrotreating (HDT) and (ii) hydrocracking (HYC) units. HDT units are commonly used to reduce the content of undesired components. Thus, hydrodesulfurization (HDS), hydrodenitrogenation (HDN), hydrodeoxygenation (HDO), and hydrodearomatization (HDA) reactions occur in the processing of light and medium distillation fractions. When heavier streams are processed together with the aforementioned reactions, hydrodemetallization (HDM) and hydrodeasphaltenization take also place. Furthermore, HYC units aim to convert heavy fractions, such as vacuum, coker, or atmospheric gas oil, into lighter fractions, that is, gasoline and diesel. HYC units may be classified into two subgroups depending on the severity of the treatment. Thus, mild hydrocracking (MHYC) and standard hydrocracking units (HYC) are available in refineries. Table 6 shows the common operating ranges of the different hydroprocessing units.

**Table 6:** Standard Operating Ranges of the Different Types of Hydroprocessing Units^215−217^

conditions	hydrotreatment (HDT)	mild hydrocracking (MHYC)	hydrocracking (HYC)
temperature (°C)	270–400	320–440	380–450
pressure (bar)	25–50	35–70	90–210
H_2_/feed (m^3^/m^3^)	300–500	300–700	1000–2000
LHSV (h^–1^)	2–4.0	0.3–1.5	0.4–2.0

This type of unit is quite extended within modern refineries. Indeed, at least three hydroprocessing units are usually installed:^218,219^ (i) one for naphtha, (ii) one or two for light gas oil, and (iii) one or two for heavy or vacuum gas oil. The units used for hydrocracking purposes are less numerous than those for hydrotreating, but the installation of hydrocracking units has increased in recent times in order to fulfill environmental policy requirements for fuels.

Metal/acid bifunctional catalysts are used in hydroprocessing. A metal function, composed of transition metals or noble metals, as detailed in Table 7, is responsible for the hydrogenation and hydrogen-transfer reactions. Conversely, the acid function (Table 7) catalyzes the cracking of the skeleton of the hydrocarbon on the Brønsted acid type sites. Nevertheless, there are relevant synergistic effects between both functions.^220^ Hence, the metallic function, apart from boosting hydrogenation reactions, promotes the cracking activity of the acid function by forming an intermediate olefin by dehydrogenation. Furthermore, the acid strength of the acid sites is a key parameter, as the ring opening reactions of aromatic compounds require very strong acid sites.^221,222^

**Table 7:** Common Metallic and Acid Functions Used in Hydroprocessing Catalysts

catalyst	use	catalytic activity
Metallic Functions		
CoMo	HDS	moderate
NiMo	HDN, MHYC	high
NiW	HDN, MHYC	very high
PtPd	HDA, HYC	high
Acid Functions		
γ-Al_2_O_3_	HDA	low
amorphous SiO_2_/Al_2_O_3_	MHYC	high
HY and HZSM-5 zeolites	HYC	very high

The main challenges that refineries need to face with regard to hydroprocessing units are^223,224^ (i) the adaptation of the product streams to legislation concerning emissions when burning the fuels and (ii) the upgrading of secondary refinery streams, which, due to their content of heavy molecules, aromatics, or heteroatoms, cannot be fed into other catalytic processes.

### 6.2 Hydroprocessing of Plastics Dissolved in Refinery Streams

The hydroprocessing of neat plastics with the aim of converting them into liquid fuels has been extensively studied in the literature.^225^ Indeed, detailed studies have been conducted on the effect of several operating conditions, including H_2_ pressure, contact time, temperature, type of catalyst, and type of polymer. However, as this review assesses the valorization of waste plastics in the refinery units, only the studies involving the hydroprocessing of waste plastics together with a refinery stream have been considered. In this line, the works published by Turkish researchers from Izmir University of Technology are worth mentioning. Karagöz et al.^226^ studied the hydrocracking of LDPE (25 wt %) in VGO using activated carbon-supported metal (Ni, Co, Mo, NiMo, and CoMo) catalysts. They concluded that the CoMo/Ac catalyst showed a good liquid yield and the best HDS performance. Furthermore, the hydrocracking with this catalyst barely increased the content of aromatics with respect to that in the feedstock (4.00 and 3.45 wt %, respectively). In a later work,^227^ these researchers studied the hydrocracking of HDPE (20 wt %) in VGO using the aforementioned catalysts. Among the activated carbon-based catalysts synthesized by them, Mo/Ac catalyst reached the highest sulfur removal, whereas Co/Ac catalyst showed the highest cracking activity. Uçar et al.^228^ studied the hydrocracking of binary and ternary blends of different polymers (20 wt %) with VGO. These binary blends consisted of LDPE/VGO and PP/VGO. In the hydrocracking of LDPE/VGO, the content of naphtha was maximized at 425 °C with a commercial catalyst and with Co/Ac catalyst at 435 °C. With regard to PP/VGO blend, the best result concerning the liquid yield and content of naphtha was obtained at 425 °C with the Co/Ac catalyst. Furthermore, ternary blends were prepared by adding PVC to the binary blends. Therefore, a previous dechlorination step was required, in which the ternary blends were subject to a pyrolysis step at 350 °C for 1 h, which led to the degradation of all the polymers. The same double-step strategy has been approached in studies involving the treatment of several steps, such as (i) a blend of PE (20 wt %), PVC (5 wt %), and HVGO,^229^ (ii) a blend of waste plastics (20 wt %) with HVGO by using red mud as a catalyst for the dechlorination step,^230^ and (iii) a blend of PVC with an atmospheric bottom residue.^231^

Based on the good results obtained from the cofeeding of single polymers with VGO, they faced the coprocessing of the plastic fraction in the MSW (20 wt %) with VGO using Co/Ac and two commercial hydrocracking catalysts.^232^ The plastic fraction from MSW was composed of the following polymers: HDPE, LDPE, PP, PVC, PS, and PET. Two different strategies were used in this case, a single-step and a double-step strategy. In the latter, the blend was submitted to a previous dechlorination step due to the presence of PVC in the mixture of polymers. Nonetheless, the dechlorination process hardly affected the results, meaning that the catalysts were not poisoned by the chlorine in the PVC. Comparing the performance of the different catalysts, these researchers concluded that Co/Ac catalyst showed the best performance concerning the liquid yield and quality of the liquid.

The hydrocracking of blends of LDPE, HDPE, PP, and PS with an atmospheric bottom residue was studied by Nahid et al.^233^ and by Siddiqui and Redhwi.^234^ Overall, these researchers determined that both reaction temperature and contact time strongly influenced the process. Thus, the conversion level reached with the PS at 430 °C for a contact time of 60 min was the highest compared with that for the remaining polymers. Moreover, PS and PP promoted the formation of hydrocarbons with a boiling point below 550 °C. Finally, they concluded that the addition of the polymers improved the conversion levels reached, which is evidence of the compatibility between the reaction mechanisms of the plastics and the refinery stream.

Ali et al.^235^ investigated the coprocessing of PP with vacuum residue (VR) and coal. In this work, a detailed catalytic study was carried out, as 14 different transition metal-based catalysts were tested. Promising results were obtained, as high yields of liquids in the boiling range of 100–480 °C were obtained, together with slight formation of gums and coke. They concluded that the hydrocracking of this ternary blend is a feasible process to convert these three low value feeds into high value liquid fuels. Indeed, this option could be of great interest for countries with high deposits of coal, such as China,^236^ as it is a more environmentally friendly way of converting coal into fuels than others used at present.

**Figure 13:**
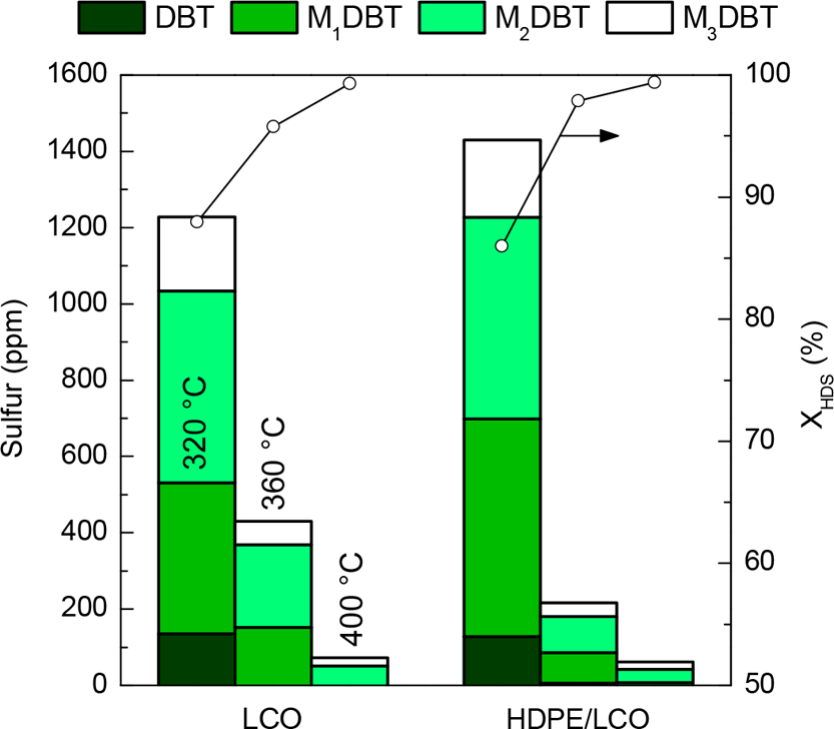
Sulfur levels obtained in the hydroprocessing of neat LCO and the blend HDPE/LCO. Adapted from the work by Palos et al.^237^

Recently, Palos et al.^237^ carried out the valorization of HDPE blended (10 wt %) with a common hydroprocessing feedstock, LCO. Their study quantified the extent of different simultaneous phenomena involved in these reactions of different nature, such as HDS, HDA, and HYC. Thus, it accounted for the changes in the content of sulfur, aromatics, and heavy molecules. Each mechanism was affected in a different way by the addition of HDPE. Hence, HYC was negatively influenced, as the content of the gas oil fraction with the cofeeding of HDPE doubled the content obtained with neat LCO. With respect to the mechanisms involving HDA, the addition of HDPE caused subtle changes, as it just increased 1 wt % the total content of aromatics. Conversely, HDS mechanisms were positively affected by the cofeeding of HDPE from 360 °C onward (Figure 13). The authors attributed this behavior to the boosting of the adsorption of sulfur-containing molecules on the active sites, with the chains of HDPE in the reaction medium. Therefore, the existence of synergistic effects between the dissolved HDPE chains and the LCO cannot be avoided. However, the conversion levels reached with the HDPE were not outstanding, as a maximum conversion of 31 wt % was obtained at 400 °C.

Subsequently, a study by Vela et al.^238^ approached the hydrocracking of a blend of HDPE (20 wt %) with VGO, but they performed a detailed analysis of the product fractions. Thus, they analyzed the composition of the gas fraction, distinguishing between dry gas and LPG fractions, as well as the composition of the gasoline and diesel fractions. Their results are evidence that the hydrocracking of the blend with the NiW/USY catalyst leads to very similar behavior as that with the VGO, and therefore the HDPE can be coprocessed without any serious problem. Moreover, 80 wt % HDPE was converted at 440 °C. Contrarily, when a PtPd/HY catalyst was used, the addition of HDPE modified the composition of the products, especially those of gasoline and diesel fractions. Thus, the gasoline fraction obtained with the blend was significantly richer in 1-ring aromatics than that obtained with neat VGO. The diesel fraction produced from the blend is more paraffinic than that from the VGO. Furthermore, HDPE conversion levels obtained with this catalyst were very high, as total conversion was obtained at 440 °C. However, a huge amount of gases were formed under these conditions, with yields being as high as 49 wt %.

Another different strategy has been also studied in the literature. It consisted in cofeeding plastic pyrolysis derivatives or waxes to hydroprocessing units. Escola et al.^239^ studied the hydroreforming of LDPE pyrolysis oil with Ni catalysts supported on hierarchical beta zeolites. After preparing catalysts with different contents of Ni (1.5–10%), the authors concluded that a content of 7% provided the best results. Thus, the production of gasoline and diesel fractions was maximized (81 wt %) with this catalyst, which also led to high hydrogenation activity. Likewise, Serrano et al.^240^ studied the hydroprocessing of the same feed on Pd catalysts supported on hierarchical ZSM-5 zeolites. They produced gasoline and diesel range hydrocarbons with acceptable contents of aromatics. Indeed, the hierarchical porous structure of their catalysts maximized the production of liquid fuels (selectivities above 95%) by reducing the overcracking reactions that lead to the production of gases and controlling the composition of obtained products.

Even though gasoline and diesel are commonly the targeted products, studies aimed at the production of jet fuel have also been conducted in the literature. Zhang et al.^241^ obtained high yields of jet fuel range hydrocarbons through a two-step process. The first process consisted of a catalytic microwave degradation of LDPE at 375 °C in the presence of a zeolite based catalyst, whereas the second step was a hydrogenation of the products leaving the first step on a Raney Ni catalyst. They concluded that the final products fit the quality standards for RJ-5 and JP-10 jet fuels and JP-5 navy fuel. Similarly, Tomasek et al.^242^ studied the hydroprocessing of PE and PP pyrolysis products blended with kerosene on a NiMoP/Al_2_O_3_. They showed that PP pyrolysis derivatives and kerosene can be converted into jet fuel in a single step, although the products of the blend formed by PE pyrolysis derivatives and kerosene required an additional hydroisomerization step to improve their quality.

Mangesh et al.^243^ studied the performance of different blends of hydrogenated propylene pyrolysis oil (HPPO) with diesel in a diesel engine. The hydrogenation of propylene pyrolysis oil was previously carried out on a Ni/HZSM-5 catalyst at 350 °C and 70 bar. These authors concluded that 10 and 20 wt % of HPPO in the blend led to a fuel complying with EN590 Standard for diesel fuels. In later works, these researchers improved the results by using Au/mordenite^244^ and NiMo/laponite^245^ catalysts in the hydroprocessing of propylene pyrolysis oil. The improvement is due to the multifunctionality of these catalysts, as they are active in hydrogenation, isomerization, and aromatization reactions.

As the viability of hydroprocessing depends on the availability of H_2_, the possibility of obtaining H_2_ by means of steam reforming of waste plastics is also interesting. Different approaches have been proposed for tackling this process, but those based on a two-step strategy of pyrolysis and reforming are noteworthy.^3,246^ Barbarias et al.^247^ used a system equipped with a conical spouted bed reactor connected in-line with a fluidized bed reactor for the steam reforming of the volatiles. In another work,^248^ these authors modeled the steam reforming step considering the deactivation of the commercial Ni catalyst. The proposed reaction scheme considered separately C_5+_, C_2_–C_4_, and methane reforming reactions and the water–gas shift reaction. At 700 °C, the values obtained for the yield and concentration of H_2_ were 85.7 wt % and 70 vol %, respectively, with a moderate catalyst deactivation for a space time of 16.7 g_cat_ min g_HDPE_^–1^. The stability of the catalyst strongly depended on the operating conditions. Thus, the stability increased with temperature, space time, and steam/plastic ratio due to the low coke deposition rate.^249^ The study of steam reforming of different plastics (HDPE, PP, PET, and PS) and their mixture revealed significant differences in the yield of hydrogen obtained.^250^ The highest yield was obtained with the polyolefins (up to 37.3 wt %), followed by the yields obtained with PS and the PET (29.1 and 18.2 wt %, respectively). They also observed important differences in the nature of the coke deposited, which was filamentous in the reforming of polyolefins and encapsulating in the reforming of PET, PS, and the mixture.^251^

### 6.3 Hydroprocessing of Tire Pyrolysis Oil

The high contents of sulfur and aromatics in the TPO constraint its direct use as fuel in internal combustion engines, and it must be subjected to hydroprocessing in order to improve its properties. Some of these studies have been carried out in slurry reactors following batch processes. Debek and Walendziewski^252^ tested commercial CoMo/SiO_2_–Al_2_O_3_ and NiMo/Al_2_O_3_ catalysts, which reduced the content of sulfur below 0.2 wt %. Similarly, Djandja et al.^253^ reduced the contents of nitrogen and sulfur to 0.09 wt % and 15 ppm, respectively, using a Pd/C catalyst and tetralin as hydrogen donor. However, the treated oil is basically aromatic, with a content of 40 wt % monoaromatics.

**Figure 14:**
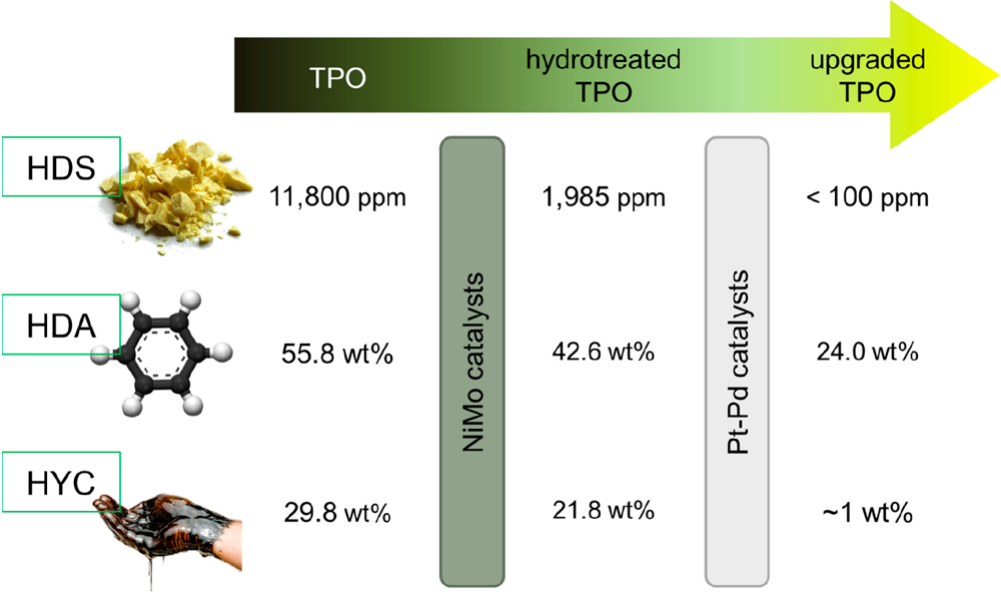
Results obtained in the hydroprocessing of neat TPO operating in a two-stage strategy. Adapted from the work by Hita et al.^71^

Hita et al.^254−257^ studied the hydroprocessing of TPO in a trickle-bed reactor in a two-stage strategy. The configuration of the strategy, together with the most relevant results, is summarized in Figure 14. They have used NiMo-based catalysts in the first step,^254^ with the main goal of reducing the content of sulfur of the TPO. Indeed, they obtained good results as they reduced the content of sulfur from 11800 ppm to values below 2000 ppm in the hydrotreated TPO. Furthermore, although they operated under mild conditions, they also reduced the content of aromatics and gas oil fraction molecules by 13.2 and 8 wt %, respectively. Nonetheless, HDA and HYC reaction conversions are significantly improved in the second step operating at more severe conditions and on a Pt–Pd/SiO_2_–Al_2_O_3_ catalyst.^256^ Hence, the higher hydrogenation activity of the noble metals and the higher acidity of the support to promote cracking reactions was clearly evidenced in the obtained results. Consequently, they obtained a reduction of 18.6 wt % in the content of aromatics, with almost no gas oil fraction molecules and sulfur contents below 100 ppm.

Later, these authors proposed a lump-based kinetic model to describe both hydroprocessing steps.^258^ In this model, they considered HDS, HDA, and HYC mechanisms for each step, that is, hydroprocessing and hydrocracking steps. Furthermore, catalyst deactivation was negligible in the first step but highly significant in the hydrocracking one. In view of this fact, catalyst deactivation was considered in the modeling of the hydrocracking step. In this context, they tested the hydroprocessing of the TPO using in-house prepared activated carbon-based catalysts. First,^255^ they studied the performance of three NiMo catalysts supported on tailored activated carbon. The activated carbon was obtained through physical activation of petroleum coke or petcoke, which is a valueless byproduct in the oil industry. Their results show that short carbon activation times and functionalization with HNO_3_ led to highly active carbon supports, especially for HDS. Furthermore, they prepared Pt–Pd catalysts supported on activated carbons for the hydrocracking of the hydrotreated TPO.^257^ This time, the material chosen for the preparation of the activated carbons was olive stone, which is a residue of the olive-oil sector. Moreover, the olive stones were impregnated with an aqueous solution of H_3_PO_4_ in order to create phosphate groups on the support that conferred strong acid sites upon it. This catalyst performed well in the removal of sulfur (97.3%), as well as in the reduction of the gas oil fraction (97%). Therefore, based on these two studies, these researchers proved that high-quality fuels can be produced by hydrotreating TPO using cheap materials as sources of activated carbons.

Liu et al.^259^ studied the hydrotreatment of a blend of the oil obtained in the hydrothermal liquefaction of EOL tires, which is a liquid similar to TPO, and used engine oil. They tested different commercial noble metal-based (Pd, Pt, Ru, Ir, and Rh) catalysts supported on activated carbon. The most relevant findings of their study are that Rh/C is the most appropriate catalyst for removing sulfur and that about 94% of the energy contained in the feeds is contained in the final product. Therefore, they proved that EOL tires together with waste engine oil can be co-converted into diesel-like fuels.

The deactivation of the precious metal-based catalysts supported on activated carbons used for the hydrocracking of TPO, both raw and hydrotreated, is mainly caused by the deposition of carbonaceous species. Thus, Cordero-Lanzac et al.^260^ studied the nature and location of the coke deposited on these catalysts in order to achieve a total recovery of the activity after each reaction–regeneration cycle. Two different types of coke were identified. The first type was correlated with light deactivating species located on the external surface of the catalyst or on the metal sites. This first type of coke was burned at temperatures below 310 °C. On the other hand, the removal of the second type of coke required temperatures of 400 °C as it is composed of more condensed structures located within the micropores of the carbonaceous support. Consequently, their removal by means of air combustion appeared quite feasible. However, they observed that the combustion profile of the support was different, which was attributed to a heavy coke fraction deposited on the activated carbon structure. Therefore, a partial recovery of the surface and acid properties could just be achieved.

Following a more realistic approach than the valorization of neat TPO, Palos et al.^171^ studied the hydrotreatment of a blend of TPO (20 wt %) and a current refinery stream (LCO). They tested the performance of three different benchmark catalysts assessing simultaneously the extent of HYC, HDA, HDS, HDN, and HDO mechanisms. Even though the catalysts had quite different features, the quality of the products obtained with all the catalysts was similar. Thus, they concluded that the heavy molecules within TPO were involved in rate-controlling steps. Nonetheless, the multiple analyses of the products that they carried out provided the following information: (i) catalysts with large metal surface, high total acidity and wide pores promoted HDS reactions; (ii) a network rich in Brønsted acid sites that are well-dispersed boosted HYC and HDA, and especially HDO reactions; (iii) strong acidity led to HDN, HYC, and HDA mechanisms.

## 7 Challenges and Perspectives

In this section, is presented an analysis of the main economic and technical aspects that impact on (i) the integration of waste plastic and EOL tire pyrolysis units in a refinery and (ii) the cofeeding of their pyrolysis liquid products (PPO and TPO, respectively) in FCC and hydroprocessing units. Likewise, in the midterm the main economic and social advantages of these initiatives within the scope of the circular economy action plan are assessed. Thus, some of barriers for the industrial implementation of these initiatives together with the prospects for their establishment are explained.

### 7.1 Economic and Technical Aspects

The recycling of waste plastics and EOL tires in a refinery results in a decrease of the oil consumption with subsequent reduction in capital expenditure on exploration and increase of the oil reserves. The production of plastics requires about 8 wt % of the oil consumed worldwide, this consumption being almost equally divided into the production of monomers and the coverage of the energy demand required for its production. Furthermore, the recycling of these wastes in a refinery would have a higher incidence, since the cofeeding of PPO and TPO to FCC and hydroprocessing units does not require the previous conditioning treatments that crude oil requires. All these treatments have high specific energy consumption, especially atmospheric and vacuum distillation units (1.16 × 10^5^ and 0.95 × 10^5^ kJ bbl^–1^, respectively).^261^

Different pilot plant scale technologies have been developed for the production of fuels from waste plastics^8,9^ and EOL tires.^21^ Nevertheless, the scale-up of these plants has several difficulties affecting their industrial implementation, the most important ones being the economic cost^262^ and the conditioning and marketing of obtained fuels. These last barriers do not exist in refineries, as their units are highly versatile, have huge refining capacity and, commonly, have been already depreciated. Hence, the high capital investments required by installations developed *ad hoc* are avoided by using refinery units. Moreover, the products would flow to the markets together with conventional fuels, requiring adapting their composition to environmental and regulatory concerns to minimize the emission of pollutants, for example, nitrogen and sulfur oxides and particulate matter. Reaching these legal requirements is really challenging for a process specifically designed and funded for the valorization of waste plastics or EOL tires. It must be taken into account that, in spite of the HHV of PPO and TPO, they are not recognized under EU legislation (Waste Framework Directive, WFD 2008/98EC) as waste recycling products because they are mainly used for energy generation purposes. Equally, the International Standard Organization (ISO 15270:2008) makes the same consideration.^8^ These restrictions are inherent to the circular economy strategy and affect the recycling of different materials that must keep to certain quality standards.^263^ Consequently, the involvement of the refineries is necessary for (i) minimizing the barriers for the production and commercialization as fuels of the products obtained in the pyrolysis of these wastes and (ii) receiving institutional incentives for contributing to circular economy transition. In particular, refineries would benefit from the reduction of taxes for the wastes-derived fuels for considering them as green fuels and by economic incentives for contributing to reduce CO_2_ emissions.

An additional setback for the industrial implementation of a waste pyrolysis plant could be the high-energy demand inherent to the endothermic nature of the process. This problem was studied by Elordi et al.,^77^ who have computed the energetic viability of a waste plastics (mixture of LDPE, HDPE and PP) pyrolysis plant with a capacity of 1000 kg h^–1^. These researchers determined that the combustion of 5 wt % of the less attractive product stream would cover the energy demand of both pyrolysis and distillation stages.

It is worth noting that according to the results discussed in sections 5 and 6, no specific catalyst is required for the cofeeding of PPO and TPO. Indeed, conventional FCC and hydroprocessing catalysts commonly used in refineries are appropriate for the cofeeding of these alternative feeds. Moreover, current trends of improving the performance of FCC and hydroprocessing catalysts are triggered by the necessity of increasing the versatility of the feeds, including PPO and TPO. Among these, the following trends for FCC catalysts stand out:^264,265^ (i) the combination of USY and HZSM-5 zeolites in order to promote the selectivity and inhibit the secondary hydrogen-transfer reactions and (ii) the agglomeration of zeolites in a matrix with meso- and macropores to attenuate catalyst deactivation. With regard to the trends in hydroprocessing, the following stand out:^266^ (i) combination of the nitrogen and sulfur removal stages with the hydrocracking ones; (ii) reduction of the deactivation of the metallic function; (iii) tailoring of the porous structure and acidity of the zeolite used as a support.

### 7.2 Social, Environmental, and Safety Aspects

Given the seriousness of the environmental impact that the inefficient management of waste plastics and EOL tires causes, the intervention of the oil industry is consistent with the social responsibility of this industrial sector. Furthermore, from an environmental point of view, the valorization of these wastes in refinery units ensures the process safety and reduces CO_2_ emissions. Indeed, the design and operation of refineries is subjected to strict safety and environmental protection regulations. Moreover, the valorization of the CO_2_ formed can be carried out at large scale, promoting the viability of the advances in CO_2_ capture, storage, and conversion into fuels.^267^

The delocalization of pyrolysis units and the centralized valorization of their liquid products in refineries is an attractive strategy to combine the pros of both approaches. In addition, this strategy combines the interests of waste collection and fuel production sectors with those of public administrations. This way, pyrolysis can be carried out in small plants located near the waste collection and segregation points, in simple and environmentally friendly units that can even be mobile. Pyrolysis liquid product could be transported to a single refinery from different pyrolysis units from a vast geographical area. Rodríguez et al.^200^ estimated that the cofeeding of 5 wt % of polyolefins with VGO to an FCC unit (average capacity of a standard unit of 50000 barrels per day) allows for valorizing about 400 tons of polyolefins per day and for saving the same amount of the current feedstock and crude oil. Furthermore, the cofeeding of that amount of plastics has no negative impact on the quality of obtained products and it will not require unconventional operating conditions on the FCC unit, the subsequent separation, nor reforming units.

In addition, for the implementation of this kind of initiative it will be necessary to adapt and coordinate the activity of different socioeconomic sectors: (i) waste collection, classification, segregation, and conditioning sector in order to include waste plastics and EOL tires in pyrolysis units; (ii) the sector in charge of the operation of pyrolysis units and conditioning and storage of liquid and solid products; (iii) the transport sector that will carry liquid products to refineries; (iv) the different enterprises that will valorize secondary products (char, CBp, and metallic components of the EOL tires). The economic viability of the pyrolysis of tires requires the valorization of the CBp.^22,23^ Furthermore, the activation of these economic sectors will require the collaboration of different administrations and can be crucial in the matter of employment generation. This way, it must be taken into account that the establishment of the circular economy strategy in glass and paper industries counts on collecting installations and with staff and means of transport commonly subsidized.

### 7.3 Perspectives

The attempt of new streams, such as PPO and TPO, in FCC and hydroprocessing units could be considered a risky operation as it can cause failures of the feeding instruments and alterations in the operation of the refinery. Consequently, the industrial attempts of new feeds require clear prospects of economic profit, quantifying the pros and cons of the implementation of this initiative. FCC units are highly versatile, available in almost every refinery, and, commonly, already depreciated units. They are periodically submitted to inspection and upgrade (every 4–5 years), the period prior to the inspection stage being the selected one to treat alternative streams. Likewise, the cofeeding of waste pyrolysis liquids would be initially tested in a refinery with small concentrations in order to determine the necessity of adapting the operating conditions. Hydroprocessing units and, in particular, hydrocracking ones have more difficulties for testing the cofeeding of PPO and TPO because (i) they are less versatile than FCC units, (ii) product distribution strongly depends on the composition of the feedstock and on selected operating conditions, and (iii) the operation of these units is very sensitive to catalyst deactivation.^268^

In addition, the valorization of waste plastics offers the possibility of recovering the monomers as another interesting goal of the fast pyrolysis stage. The fast pyrolysis of polyolefins offers good results regarding the obtained yield and selectivity operating with^123^ and without^122^ catalysts and from polystyrene^85^ and poly(methyl methacrylate).^86^ Thus, the monomer recovery can be a complementary strategy to the valorization of the PPO in FCC and hydroprocessing units. On the other hand, the aromatic nature and high content of sulfur in TPO make difficult its valorization. Thus, fuel production from TPO will require the integration of the FCC and hydroprocessing units and an additional final hydrocracking stage under severe conditions. Furthermore, the valorization of the CB obtained will be crucial in the economic balance of the pyrolysis of tires.^22,153^

## 8 Conclusions

The increasing generation of waste plastics and EOL tires, together with the lack of economical and environmentally friendly solutions for their removal, demand rational solutions for the upgrading of high-value added materials within these wastes. These solutions must comply with the restrictions in force concerning energy products, which require the adaptation of their composition in order to be used as fuels or raw materials. The physical and chemical treatments to be used to meet the required levels must be implemented on a large scale to be economically profitable. In the end, the different waste valorization initiatives proposed are commonly hindered due to scarce capital investment. Nevertheless, these treatments can be carried out in already depreciated units commonly available in the oil industry.

The experimentation under conditions similar to the industrial ones has revealed that fluid catalytic cracking (FCC) and hydroprocessing units are the best positioned candidates for the large-scale valorization of waste plastics and EOL tires. Indeed, these processes allow obtaining automotive-like fuels (gasoline and diesel) and the recovery of the monomers. The research results obtained in this field are highly encouraging and have shown that the best strategy would consist of blending these wastes in low concentrations with the current refinery streams. This way, waste plastics and EOL tires would be valorized without causing any significant impact on the quality of the products obtained or on the required operating conditions. Furthermore, based on the great capacity of FCC and hydroprocessing units, the proposed cofeeding would cover the wastes produced in large geographical areas.

The cofeeding of polyolefins or of their pyrolysis derivatives (PPO) dissolved in the current feedstock of FCC units (VGO) has notably positive synergistic effects on the yield and composition of obtained gasoline fraction. The valorization of EOL tire pyrolysis oil (TPO) is more effectively done in hydroprocessing units, cofeeding it together with other aromatic refinery streams with high contents of sulfur, such as LCO, which require similar hydroprocessing conditions.

The involvement of the oil industry in the waste recycling chain would not require any modification in their production strategy or in the implementation of new units within the refinery complex. This way, the pyrolysis of EOL tires and waste plastics in delocalized units would allow supplying homologated liquid streams with homogeneous and controlled composition to refineries. Furthermore, associated employment may be created around these environmentally friendly small pyrolysis units, as it would be required for collection, segregation, and recycling of consumer society wastes, which means economic and social impact in the surroundings.

In short, the waste refinery is an initiative that aims at the integration of the chemical industry, especially the oil industry, in the waste recycling chains. The main goal consists of the resolution of one of the major current environmental issues, which is the inability to manage the amount of waste produced daily. The proposed strategy would create a new and coherent business network for the collection and treatment of wastes. The business network would also involve the oil industry in the sustainable development, with the benefits being as follows: (i) the public opinion about refineries would definitely be improved; (ii) the availability of their raw materials would increase; (iii) tax deductions may be applied to refineries for reducing the net amounts of CO_2_ emitted to the atmosphere and for contributing to preservation of the environment. Furthermore, this initiative would be a step forward in the continuous adaptation of refinery units to alternative feeds, which could be refinery streams (VGO or LCO) or new feeds, such as tar sands, bio-oil, and wastes.

## Abbreviations

BD: butadiene
BMEP: brake mean effective pressure
BTX: benzene, toluene, and xylenes
CB: carbon black
CBp: adulterated carbon black
CSBR: continuous spouted bed reactor
EA: ethyl acrylate
EOL: end-of-life
FBP: final boiling point
FCC: fluid catalytic cracking
HCO: heavy cycle oil
HDA: hydrodearomatization
HDM: hydrodemetallization
HDN: hydrodenitrogenation
HDO: hydrodeoxygenation
HDPE: high-density polyethylene
HDT: hydrotreating
HDS: hydrodesulfurization
HHV: high heating value
HPPO: hydrotreated plastic pyrolysis oil
HYC: hydrocracking
IBP: initial boiling point
IPW: industrial plastic waste
LCO: light cycle oil
LDPE: low-density polyethylene
LLDPE: linear low-density polyethylene
LPG: liquefied petroleum gas
MAT: microactivity test
MHYC: mild hydrocracking
MMA: methyl methacrylate
MP: microplastics
MSW: municipal solid waste
NP: nanoplastics
PAH: polyaromatic hydrocarbon
PC: polycarbonate
PCPW: postconsumer colored plastic waste
PE: polyethylene
PET: poly(ethylene terephthalate)
PMMA: poly(methyl methacrylate)
PONA: paraffins, olefins, naphthenes, and aromatics
PP: polypropylene
PPO: plastic pyrolysis oil
PS: polystyrene
PVC: polyvinyl chloride
PWPW: postconsumer white plastic waste
REO: rare earth oxide
RON: research octane number
TPO: tire pyrolysis oil
VGO: vacuum gas oil
VOC: volatile organic compound
